# Phase 1 study of chidamide in combination with venetoclax, azacitidine, aclarubicin, cytarabine and G-CSF for refractory/relapsed acute myeloid leukemia: clinical safety, efficacy, and correlative analysis

**DOI:** 10.3389/fimmu.2025.1698710

**Published:** 2025-12-11

**Authors:** Yanan Wen, Jingjing Yang, Xiawei Zhang, Qingyang Liu, Wenjing Gao, Lili Dong, Weijia Zhao, Sai Huang, Daihong Liu, Yu Jing, Liping Dou

**Affiliations:** 1 Medical School of Chinese PLA, Beijing, China; 2 State Key Laboratory of Experimental Hematology, Senior Department of Hematology, The Fifth Medical Center of Chinese PLA General Hospital, Beijing, China

**Keywords:** acute myeloid leukemia, relapsed, refractory, salvage regimen, venetoclax

## Abstract

**Background:**

Relapsed or refractory AML (R/R-AML) remains a particularly adverse population necessitating improved therapeutic strategies. In this phase 1 study, we evaluated the efficacy and safety of chidamide, azacitidine, cytarabine, aclarubicin and granulocyte colony-stimulating factor (CACAG) in combination with the B cell lymphoma-2 inhibitor venetoclax (VEN) in R/R-AML.

**Methods:**

We conducted a phase 1 trial to assess the safety and efficacy of CACAG-VEN regimen as a salvage induction regimen for patients with R/R AML. The primary endpoint was the treatment-related adverse events and overall response rate (ORR) following one cycle of the CACAG-VEN regimen. We also performed single-cell RNA sequencing on eight samples of bone marrow from four patients before and after CACAG-VEN treatment.

**Results:**

From January 10, 2022, to June 8, 2024, the median follow-up was 461 days (range: 180–985 days). Thirty-four patients with refractory (n = 17) and relapsed (n = 17) AML were enrolled. The ORR was 76.5%, and the complete response rate was 73.5%. In patients with composite complete response (CRc), 44% of patients attained a measurable residual disease negative status, the 1-year overall survival (OS) rate was 82.3% (95% CI: 67.8–99.9%) and the progression-free survival rate (PFS) was 79.8%. After one cycle of CACAG-VEN, 44.1% (n = 15) of patients developed grade 3–4 myelosuppression. The median durations of neutropenia and thrombocytopenia were 17 days (95% CI: 15–22 days) and 24 days (95% CI: 22–41 days), respectively. Single-cell RNA sequencing revealed that post-treatment downregulation of MCL1, HIF1A, and ABCC1, highlighting the multi-targeted action of the regimen. Furthermore, treatment response was associated with the suppression of mitochondrial activity and the activation of the p53 signaling pathway.

**Conclusion:**

In patients with R/R AML, the CACAG-VEN regimen resulted in significant clinical benefits, with a high CRc rate and encouraging survival, as well as being well tolerated.

**Clinical Trial Registration:**

https://www.chictr.org.cn/, identifier, ChiCTR2200065634

## Background

Acute myeloid leukemia (AML) is a heterogeneous and aggressive hematopoietic malignancy. Despite great advances in targeted therapy, chemotherapy, and hematopoietic stem cell transplantation (HSCT), up to 35–45% of patients are refractory to conventional 3 + 7 intensive therapy or relapse (1–4). The prognosis of refractory/relapsed (R/R) AML is dismal, with a median overall survival (OS) of 3–7 months (5). Currently, there is no standard salvage therapy for R/R AML, indicating an urgent need for novel treatment to improve the outcomes (5–8).

Venetoclax is a potent, selective inhibitor of BCL2, an antiapoptotic protein. Venetoclax binds directly to the BCL2 homology domain 3 (BH3)-binding groove of BCL2, displacing BH3 motif-containing pro apoptotic proteins, such as BCL2L11, to initiate mitochondrial outer membrane permeabilisation, caspase activation, and pro gram med cell death (9–11). Resistance to venetoclax (VEN) is mediated by other pro-survival proteins, such as myeloid cell leukemia 1 (MCL1) and B cell lymphoma extra-large (BCL-XL) (12). DNA hypomethylating agents (HMAs), such as azacitidine (AZA) and decitabine (DAC) are commonly used to treat myeloid malignancies by inhibiting DNA methyltransferases. HMAs might synergistically inhibit MCL1 and BCL-XL, thereby increasing the dependence of leukemia cells on BCL-2 (12). Recent studies have shown that low-intensity chemotherapy, including HMAs or LDAC, in combination with VEN is a viable salvage option, even in multiply relapsed/refractory patients with AML (13). Aldoss et al. assessed the efficacy of combining VEN and HMAs in 90 patients with R/R AML, achieving a response rate of 46% (14, 15). However, 30–45% of patients with R/R AML or who are unfit and have AML still fail to achieve complete remission with a low-intensity regimen (16). It is important to explore effective and safe treatment options.

Aclarubicin is an oligosaccharide anthracycline antibiotic that exerts its antineoplastic effect by intercalating into DNA and inhibiting topoisomerase, thereby disrupting DNA replication and repair. The CAG regimen, which combines Aclarubicin with low-dose cytarabine (Ara-C) and G-CSF, is designed to sensitize leukemia cells by using G-CSF to drive them into the cell cycle, thereby enhancing their susceptibility to the cytotoxic drugs. A previous study showed that combining HMAs with the CAG (cytarabine, aclarubicin and G-CSF) regimen was well tolerated and improved prognosis in elderly patients with high-risk AML (17, 18). We previously used the CAG regimen along with chidamide and decitabine (CDCAG) for R/R AML (19). The CDCAG regimen was well tolerated and showed moderate anti-leukemic activity with a complete response rate or complete response with incomplete blood count recovery (CR/CRi) rate of 46.2%, overall response rate (ORR) of 54.8%, median overall survival (OS) of 266 days, and 1-year OS rate of 36.9%. In accordance with the clinical trial results, our preclinical data showed that the histone deacetylase (HDAC) inhibitor chidamide can increase the sensitivity to anthracyclines via regulation of the HDAC3-AKT-P21-CDK2 signaling pathway in R/R AML cells (20). A multi-center study of VEN combined with the 3 + 7 regimen in newly diagnosed AML achieved a CR rate of 91% (21). These results are encouraging, indicating that epigenetic modifiers or BCL2 inhibitor combined with cytotoxic agents may represent a promising treatment direction for patients with R/R AML.

These data provide a strong clinical rationale for the combination of chidamide, azacitidine, cytarabine, aclarubicin and granulocyte colony-stimulating factor (CACAG) in combination with VEN for the treatment of R/R AML. Therefore, we conducted a single-center phase 1 trial to investigate the efficacy and tolerability of the CACAG-VEN regimen for patients with R/R AML and performed single-cell RNA sequencing using bone marrow samples from four patients before and after CACAG-VEN treatment. Our research is the first to evaluate the efficacy and side effects of VEN combined with HMAs, HDAC inhibitors, and the CAG regimen.

## Materials and methods

### Patients

In this single-center phase 1 study, we evaluated the efficacy and safety of the CACAG-VEN regimen in patients with R/R AML (ChiCTR2200065634). Patients with R/R AML (aged 14–75 years) who had an Eastern Cooperative Oncology Group performance status of 0–2 were eligible for this trial. Refractory AML was defined as no complete response [CRc, complete response (CR) plus complete response with incomplete blood count recovery (CRi)] and a reduction in bone marrow (BM) blast count of 50% after one cycle or no CRc after two cycles (22, 23). Relapsed AML was defined as recurrence of blasts in the peripheral blood (PB), BM blasts ≥ 5%, or development of extramedullary disease after achieving a CRc. Details regarding patient inclusion and exclusion criteria are shown in **Supplementary Table 1**. This trial was approved by the Ethics Committee of PLA General Hospital (Approval Number: S2022-240-01). Signed informed consent was obtained from all patients or their respective guardians before treatment. From January 10, 2022, to June 8, 2024, 34 patients were enrolled at the Chinese PLA General Hospital (**Supplementary Table 2**).

### Study design and treatment regimen

All patients in this study were treated with the CACAG-VEN regimen over a 28-day cycle: VEN was administered orally (PO) on days 1–14 (100 mg for day 1, 200 mg for day 2, 400 mg for days 3–14); chidamide 30 mg was administered orally on days 1, 4, 8, and 11; azacitidine 75 mg/m^2^ was administered subcutaneously on days 1–7; cytarabine 75–100 mg/m^2^/d was administered on days 1–5, intravenously; aclarubicin 10 mg/m^2^/d was administered on days 1, 3, and 5; and granulocyte colony-stimulating factor (G-CSF) 300 μg/day was administered until WBC > 20 × 10^9^/L. Following the initial occurrence of severe infectious events, in consultation with the DSMB to mandate enhanced supportive care measures, include mandatory antibacterial, antifungal, and antiviral prophylaxis for all subsequent patients, as well as stricter criteria for the initiation of subsequent treatment cycles. G-CSF and blood product transfusions were administered at the investigator's discretion to manage myelosuppression and its complications. BM assessment for response was performed on day 28 after starting treatment. Morphologic, cytogenetic, and measurable residual disease (MRD) assessments were performed during each BM assessment.

The use of antifungal prophylaxis was mandated per the 2022 ELN AML guidelines due to the high risk of invasive fungal infections during intensive induction therapy (3). In recognition of the significant drug-drug interaction between this strong CYP3A4 inhibitor and VEN, the VEN dose was adjusted according to the prescription information recommendations. This dose modification was a key strategy to mitigate the risk of excessive myelosuppression. Supportive treatment including aggressive hematologic support with growth factors and blood product transfusions was permitted at the investigator’s discretion.

### Dose escalation and determination

Patients were assigned sequentially to dose-escalation groups according to a 3 + 3 design, in which groups that received sequentially higher doses were opened after three patients had completed 3 weeks of treatment at the preceding dose without having dose-limiting toxic effects (DLTs). DLTs were defined as the following events occurring within the DLT evaluation period: grade≥4 nonhematologic toxicity; absolute neutrophil count (ANC) < 500/μL (grade 4) or platelets < 25,000/μL (grade 4) for > 14 days off therapy without evidence of leukemia (< 5% blasts) in the BM or blood, or > 42 days from therapy initiation, whichever is longer. The first dose was the fixed-dosed CACAG regimen and 7 days VEN (100 mg for day 1, 200 mg for day 2, 400 mg for days 3–7). Based on the acceptable safety profile in the first three patients led to the introduction of stepwise intrapatient increases in dose to the fixed-dosed CACAG regimen and 14 days VEN (100 mg for day 1, 200 mg for day 2, 400 mg for days 3–14) for the subsequent dose-escalation and expansion cohorts. The regimen containing the full 14-day course of VEN in combination with the fixed-dose CACAG was established as the Maximum Tolerated Dose (MTD). The dose-escalation data were summarized in **Supplementary Table 8**.

### Cytogenetic and molecular analysis

Molecular and cytogenetic analysis was performed centrally by G- and R-banding analyses and next-generation sequencing (NGS) using PB or BM. Molecular analysis was performed before the start of chemotherapy, at the time of relapse, and at the time of refractory disease. For patients with molecular mutations, MRD was assessed in BM specimens using mutation-specific real-time quantitative polymerase chain reaction (qRT-PCR) for NPM1, AML1-ETO, CBFB/MYH11, JAK2 V617F, MLL rearranged, PML/RARA, or NGS-based MRD detection for any other MRD marker, as reported previously (24–27). MRD assessment was performed on day 28 after the start of treatment.

### Response assessment

The primary endpoint was the treatment-related adverse events and the ORR after one cycle of CACAG-VEN treatment. The secondary endpoints included CRc rate, partial response (PR) rate, no response (NR) rate, MRD negative rate after one cycle, OS, progression-free survival (PFS), duration of response (DOR), cumulative incidence of relapse. The response criteria were defined per the European LeukemiaNet (ELN) 2022 guidelines. The main definitions are as follows: CR was defined as the disappearance of signs and symptoms of leukemia, absence of leukemic cells in the leukocyte classification, < 5% primitive cells in the BM, absence of extramedullary leukemia, and neutrophil count ≥ 1.0 × 10^9^/L, PLT ≥ 100 × 10^9^/L; a CRi was defined as the disappearance of signs and symptoms of leukemia, absence of leukemic cells in the leukocyte classification, < 5% primitive cells in the BM, absence of extramedullary leukemia, and neutrophil count < 1.0 × 10^9^/L or PLT < 100 × 10^9^/L; PR was defined as a decrease of BM primitive cells to 6–20% and decreased over 50% from pretreatment; NR was defined as non-fulfillment of the above criteria; MRD was assessed by flow cytometry. MRD was evaluated with a sensitivity of 0.01–0.10%, defining MRD negative as < 0.1% and MRD positive as 0.1% or higher; early relapse was defined as the presence of > 5% BM primitive cells or extramedullary infiltrates after achieving a CR within 12 months, whereas late relapse was defined as relapse occurring beyond 12 months; refractory disease was defined as failure to achieve CR/CRi after one or more course of induction chemotherapy regimen; OS was defined as the time from enrollment into CACAG-VEN therapy until death from any cause or last follow-up; PFS was defined as the time from enrollment until relapse or death from any cause, whichever occurred first; the ORR was calculated as CRc+PR (28); and the DOR was defined among responders as the duration between the date of response and the date of disease relapse or death from any cause, whichever occurred first.

The safety assessment primarily included an examination of the occurrence of adverse events (AEs) occurring within 30 days after the start of CACAG-VEN treatment, with determinations made through clinical symptoms, laboratory tests, and imaging examinations. The AE severity grading standard was adopted from the National Cancer Institute (NCI) adverse event grading (CTC-AE) version 4.03. Causality for all serious adverse events (SAEs), including fatal events, was assessed by the principal investigator and study clinicians using the Naranjo algorithm. This assessment systematically considered factors such as the temporal relationship to drug administration, known pharmacological effects of the regimen, the patient's underlying clinical condition, and the presence of alternative causes. Outcomes were categorized as 'definite,' 'probable,' 'possible,' or 'doubtful' in relation to the study treatment. A summary of this assessment for key SAEs is provided in **Supplementary Table 7**. An independent Data Safety Monitoring Board (DSMB) was involved for this trial. The DSMB reviewed accumulating safety data, including patients’ screening, all SAEs and DLTs, after each cohort was enrolled and prior to dose escalation. The DSMB provided recommendations to the study sponsors regarding the continuation, modification, or termination of the trial.

### Single-cell sequencing

For scRNA-seq analysis, bone marrow mononuclear cells (BMMCs) from four consecutively enrolled patients (July–September 2023) were processed using the 10x Genomics platform. Patients selected for scRNA-seq analysis were consecutively enrolled during the study period to minimize selection bias. Their diverse clinical outcomes provide a valuable spectrum for correlating transcriptomic changes with treatment efficacy. The clinical background of each patient selected for sequencing was shown in **Supplementary Table 9**. BM cells from patients with AML were collected in EDTA tubes and diluted 2:1 in ice-cold FACS buffer. Mononuclear cell separation was accomplished using density centrifugation media (Ficoll-Paque; GE Healthcare Life Sciences) at a 1:1 ratio with BM cells. Subsequently, the cell suspension, with a concentration ranging from 700 to 1,200 cells/µl and a cell viability exceeding 85%, was loaded onto the Chromium Single Cell Controller (10× Genomics) to generate single-cell gel beads in the emulsion, following the manufacturer’s protocol and employing the Chromium Single Cell 3' Library and Gel Bead Kit V3.1 (10× Genomics, PN1000268). The libraries were sequenced on an Illumina NovaSeq 6,000 sequencer.

To ensure data quality, an initial assessment of the sequenced reads was conducted using FastQC. Subsequently, FASTQ sequenced files were aligned to the human reference genome using CellRanger, and the transcript expression of each cell was quantified. The resulting expression matrix from all samples was imported into R (v4.3.2) and further analyzed using Seurat (v4.3.2).

Cells with unique feature counts exceeding 9,000 or falling below 200, and those with mitochondrial counts exceeding 15%, were excluded. Genes detected in at least three cells were retained for subsequent analysis. The combined dataset was normalized using Seurat’s “NormalizedData” function, applying a global-scaling normalization method referred to as “LogNormalize,” and then multiplied by a default scale factor of 10,000. Data integration and batch effect removal were performed using Seurat’s “RPCA” method.

Dimension reduction analysis was conducted using Seurat’s “RunPCA” function, followed by nonlinear dimensional reduction using the “RunUMAP” function. Stemness and lineage priming scores for hematopoietic stem cells and granulocyte-macrophage progenitors (HSCs/GMPs) were computed using the Seurat package’s “AddModuleScore” function. Radar plots were used to visualize the average HSC/GMP subpopulation scores at different time points.

Differential analysis to identify differentially expressed genes (DEGs) between distinct clusters was conducted using the “FindAllMarkers” function in Seurat. Gene ontology (GO) and Kyoto Encyclopedia of Genes and Genomes (KEGG) enrichment analyses were performed using ClusterProfiler for DEGs identified within each cluster.

### Sample size

The sample size was calculated according to the primary endpoint (ORR) of the study. Our previous study reported that the ORR of patients with R/R AML treated with the CDCAG regimen was 46.2% (19). Additionally, several clinical trials revealed that the ORRs for adult patients with R/R AML receiving VEN along with intensive chemotherapy (FLAG-IDA) were 72–75% (22). Therefore, an expected ORR of 75% was established for patients treated with the CACAG-VEN regimen. A sample size of 27 achieves 90% power to detect differences using a two-sided exact test with a significance level (alpha) of 0.05 (PASS software, NCSS LCC, USA). Allowing a drop-out rate of 20%, 34 patients were required.

### Statistical analysis

The patient status was determined through telephone follow-ups and the review of outpatient or inpatient records. Continuous data are described as the median with range or mean and standard deviation (SD) according to the normality of the distribution. Categorical data are described as n (%). The ORR, CR, CRi, CRc, and MRD-negative rates were calculated using 95% confidence interval (CI). The Kaplan–Meier method was used to estimate the DOR, PFS, and OS. The cumulative incidence of relapse was estimated using a competing risk model. Death without relapse was defined as a competing event for relapse. Safety analysis was used to calculate the frequency of various events and the ratio of the severity of each event by detailing the cases of blood toxic or nonblood toxic reactions. A two-sided *P* value < 0.05 was considered to indicate statistical significance. All statistical analyses were performed using Easy R (version 1.61) and R (version 4.3.3).

## Results

### Adverse events

The CACAG-VEN regimen was generally well tolerated in patients with R/R AML. One patient (2.9%) experienced early death due to infectious shock on day 19, which occurred 4 days after completing the full treatment course. This event was determined to be unrelated to the study therapy by the DSMB and attributed to the patient’s underlying leukemia, as it was associated with a pre-existing bloodstream infection. No other patients discontinued treatment due to non-fatal adverse events. For three patients received CACAG and 7-days VEN, the duration of neutropenia was 9 days, 13 days and 17 days, respectively. The duration of thrombocytopenia was 18 days, 15 days and 19 days, respectively. Apart from hematologic toxicities, patients received CACAG and 7-days VEN developed the grade 1–2 AEs of vomiting and hypokalemia. For all enrolled patients who achieved a response, the median duration of neutropenia and thrombocytopenia (ANC < 500/mL and platelets < 100) was 17 days (95% CI: 15–22) and 24 days (95% CI: 22–41), respectively. The median number of units of red cells and platelets transfused was 8.5 and 6.5, respectively. Apart from hematologic toxicities, 15 patients (44.1%) developed the grade 3 AEs of pneumonia (14.7%), hyponatremia (11.8%), hypokalemia (14.7%), and hypocalcemia (2.9%) (**Table 1**).

**Table 1:** Non-hematologic adverse events (n = 34).

Adverse events (AE)	Any grade AE, n (%)	Grade 3/4 AE, n (%)
Any AE	34 (100.0%)	15 (44.1%)
Pneumonia	10 (33.3%)	5 (14.7%)
Liver enzymes	14 (46.7%)	0 (0.0%)
Nausea /vomiting	2 (6.7%)	0 (0.0%)
Diarrhea	4 (13.3%)	0 (0.0%)
Oral ulcer	1 (3.3%)	0 (0.0%)
Urinary tract hemorrhage	3 (10.0%)	0 (0.0%)
Drug-induced kidney injury	2 (6.7%)	0 (0.0%)
Hypoalbuminemia	12 (40.0%)	0 (0.0%)
Hyponatremia	7 (23.3%)	4 (11.8%)
Hypokalemia	22 (73.3%)	5 (14.7%)
Hypocalcemia	16 (53.3%)	1 (2.9%)

### Patient characteristics

**Figure 1:**
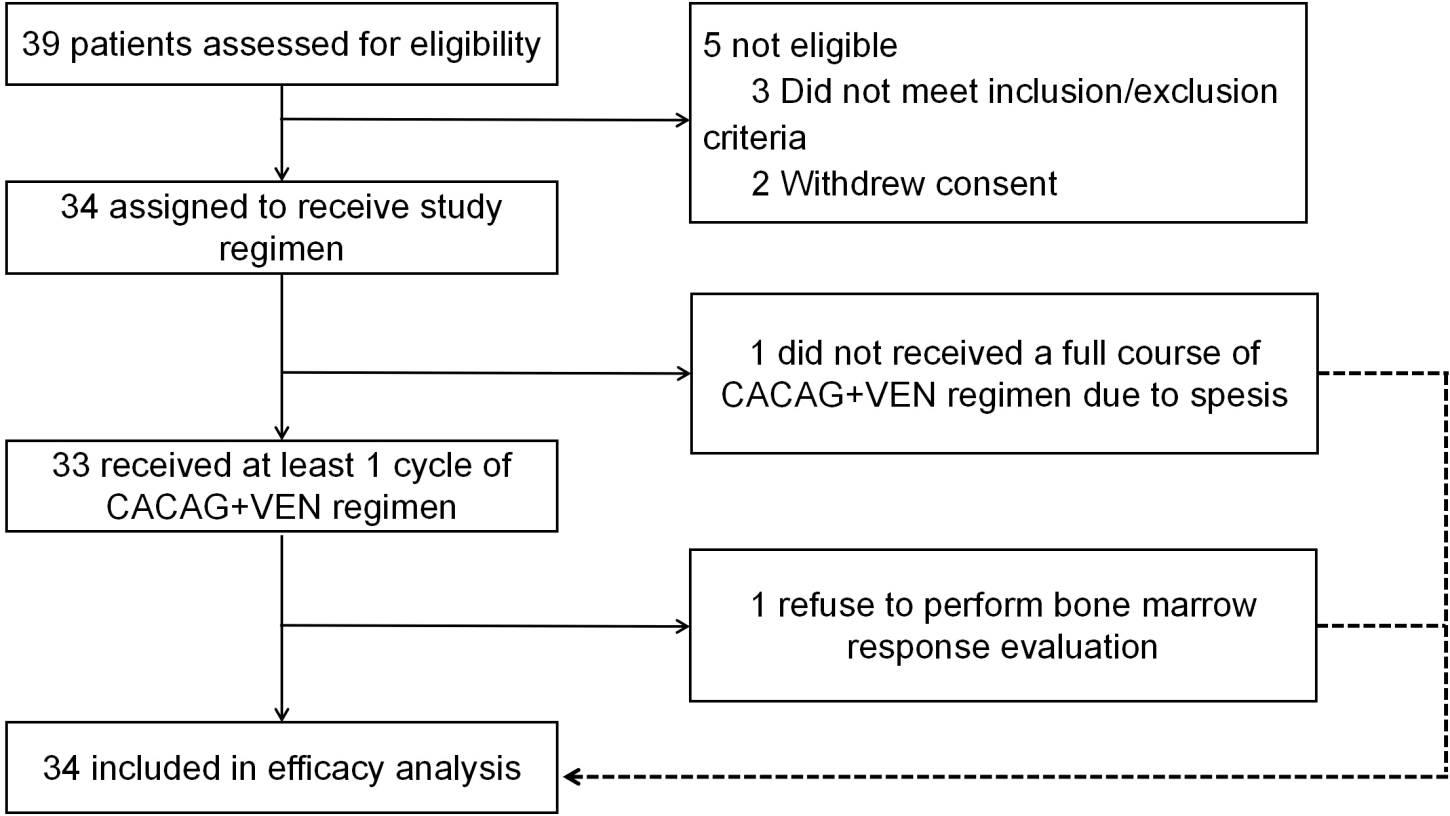
Trial profile.

The follow-up cutoff date was June 8, 2024. From January 2022 to June 8, 2024, 39 patients were screened, and 34 eligible patients were enrolled and treated with the CACAG-VEN regimen (**Figure 1**). Of the five patients who were not eligible, three patients did not meet the inclusion/exclusion criteria and two patients withdrew consent. The baseline characteristics and details of the enrolled patients are shown in **Table 2**; **Supplementary Table 2**. The median age of the participants was 45.5 years (range: 15–74 years). Of the enrolled patients, 17 patients had refractory AML (50%), ten (29.4%) had early relapsed AML (relapsed with an initial CR duration of < 12 months), and seven (20.6%) had late relapsed AML (relapsed with an initial CR duration of > 12 months). Of the 17 patients with relapse, three (17.6%) received one or two salvage regimens, and 14 (82.4%) received no salvage regimen between the last relapse and enrollment. Two patients experienced relapse of AML after hematopoietic stem cell transplantation (HSCT). Of the 17 refractory patients, 11 patients (64.7%) received one prior regimen, two patients (11.8%) received two prior regimens, and four patients (23.5%) received three or more prior regimens. Among all patients, the standard “3+7” was used previously in 26 patients (76.5%), HMAs, including azacitidine (AZA) or decitabine (DAC), were used previously in 20 (58.8%) patients, VEN was used previously in 13 (38.2%) patients, HMAs combined with VEN was used previously in 12 (35.3%) patients, and chidamide was used previously in four (11.8%) patients.

**Table 2:** Baseline patient characteristics.

Patient characteristics	Patients (n = 34)
Disease status, n (%)	
Refractory	17 (50)
Early relapse	10 (29.4)
Late relapse	7 (20.6)
Age, years (range)	45.5 (15–74)
Sex, n (%)	
Female	15 (44.1)
Prior SCT, n (%)	2 (5.9)
Prior therapy lines, n (%)	
Prior HMAs	20 (58.8)
Prior VEN	13 (38.2)
Prior HMAs+VEN	12 (35.3)
Prior Chidamide	4 (11.8)
Prior “7+3”	26 (76.5)
Prior number of therapies, n (%)*	
0	14 (41.2)
1	13 (38.2)
2	3 (8.8)
≥ 3	4 (11.8)
ECOG performance status, n (%)	
0	1(2.9)
1	17 (50.0)
2	16 (47.1)
ELN 2022 risk status**, n (%)	
Favorable	6 (17.6)
Intermediate	10 (29.4)
Adverse	18 (53.0)
Karyotype, n (%)	
Complex karyotype***	4
Baseline parameters, median (range)	
WBC.1 × 10^9^/L	2.79 [0.63–142.82]
Hb, g/L	74 [45–150]
Platelets,1 × 10^9^/L	41.5 [6–316]
BM blasts (%)	38 [4.4–93.2]
Genetic abnormalities, n(%)	
*AML1-ETO*	3(8.8)
*KMT2A* rearrangement	6(17.6)
Molecular mutations, n(%)	
*DNMT3A*	7 (20.6)
*NRAS*	5 (14.7)
*ASXL1*	4 (11.8)
*FLT3-ITD*	4 (11.8)
*IDH1*	4 (11.8)
*TP53*	4 (11.8)
*KIT*	4 (11.8)
*IDH2*	4 (11.8)
*KRAS*	3 (8.8)
*TET2*	3 (8.8)
*BCOR*	3 (8.8)
*KMT2A*	3 (8.8)
*FLT3-TKD/CEBPA/GATA2/SRSF2/STAG2/*	18 (52.9)
*CREBBP/NPM1/RUNX1/WT1^a^*	11 (32.4)
*ZBTB7A/MYC/NF1/PHF6/SETD2/DDX41/*	
*IGLL5/PTPN11/ZFHX4/TYK2/ARIDIB^b^*	

*14 relapsed patients received no salvage regimen before enrollment.

**ELN status was assessed when AML was diagnosed.

***Complex karyotype was defined as ≥ 3 clonal chromosomal abnormalities.

VEN, Venetoclax; HMAs, Hypomethylating agents; ECOG, Eastern Cooperative Oncology Group; ELN, European LeukemiaNet.

a Two patients carried with each mutation.

b One patient carried with each mutation.

### Clinical efficacy

**Figure 2:**
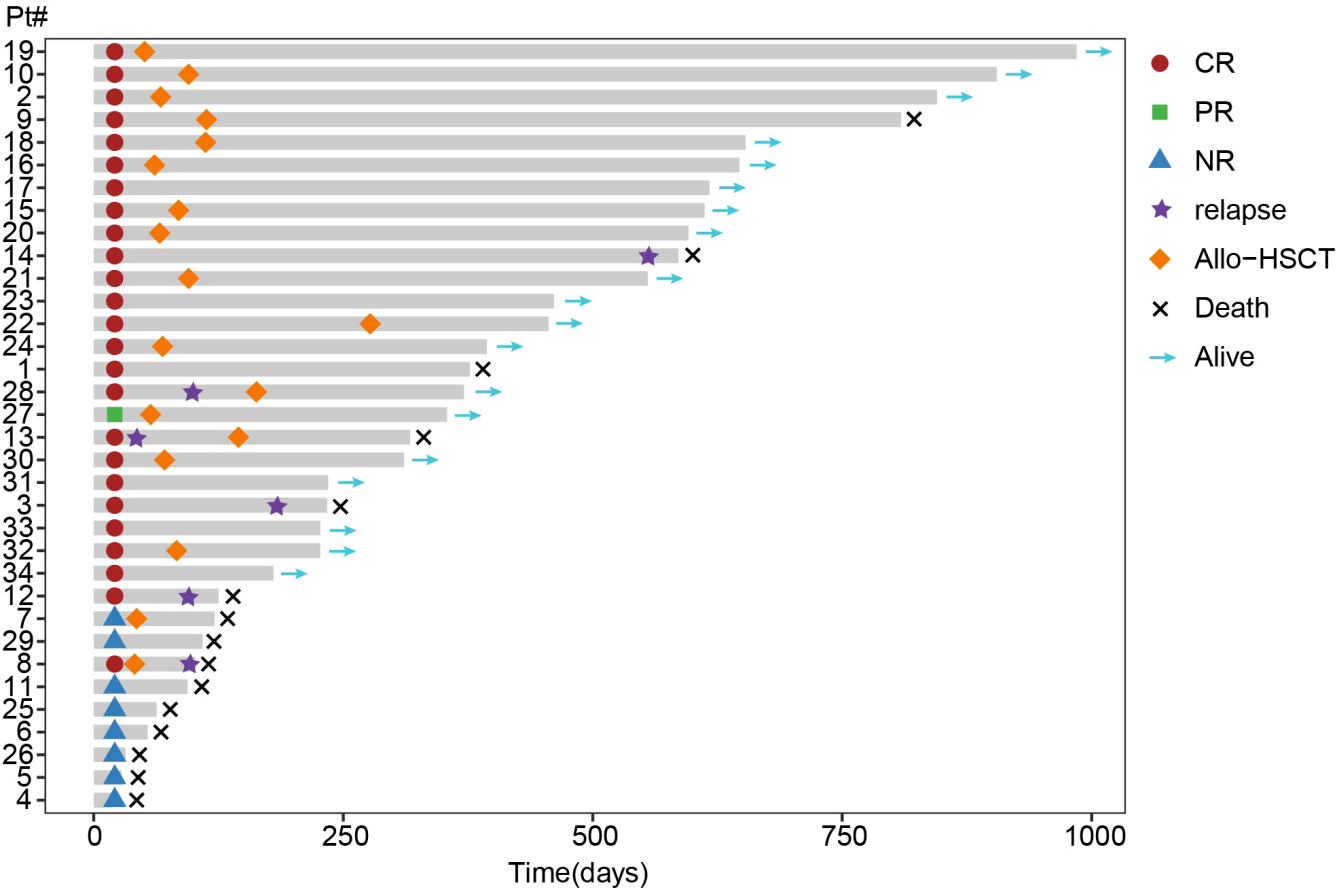
Swimmer plot of dynamic response assessment.Each bar represents an individual patient. AML: Acute myeloid leukemia, HSCT: Hematopoietic stem cell transplantation, MRD: Measurable residual disease, CR: Complete response, NR: No response, PR: Partial response.

All three patients received CACAG and 7-days VEN achieved a CRc. Among all enrolled patients, one did not complete a full course of treatment due to sepsis on day 4, and another declined BM response evaluation and died on day 19; both patients were classified as NR. After one cycle of the CACAG-VEN regimen, 26 of 34 patients (76.5%, 95% CI: 58.8–89.3%) achieved an ORR, including 25 patients (73.5%) who achieved a CRc (CR: 64.7%; CRi: 8.8%) and one who achieved a PR (2.9%). The treatment responses are summarized in **Table 3**, **Figure 2**.

**Table 3:** Response after one cycle of CACAG-VEN therapy (n = 34).

Clinical response	n, (%, 95%CI)
ORR	26 (76.5, 58.8–89.3)
CR	22 (64.7, 46.5–80.3)
CR_MRD+_	13 (59.1, 36.4–72.2)
CR_MRD-_	9 (40.9, 20.7–63.6)
CRi	3 (8.8, 1.9–23.7)
CRi_MRD+_	1 (33.3, 0.8–90.6)
CRi_MRD-_	2 (66.7, 9.4–99.2)
PR	1 (2.9, 0.07–15.3)
NR	8 (23.5, 10.7–41.2)

CR, Complete response; CR_MRD+,_ Complete response with measured residual disease (MRD) positive; CR_MRD_-, Complete response with MRD negative; Cri, Complete response with incomplete blood count recovery; CRi_MRD+,_ Complete response of MRD positive with incomplete blood count recovery; CRi_MRD-,_ Complete response of MRD negative with incomplete blood count recovery, MRD was assessed by flow cytometry; evaluated with a sensitivity of 0.01–0.10%; PR, Partial response; NR, No response.

A CRc rate of 64.7% (11/17; CR: 58.8%; CRi: 5.9%) was observed in patients with primary refractory disease and 82.4% (14/17; CR: 70.6%; CRi: 11.8%) in those with relapsed disease. A CRc rate of 70% (7/10, 5 CR and 2 CRi) was observed in patients with early relapse, whereas a CR rate of 100% (7/7) was observed in patients with late relapse. An ORR rate of 85.7% (12/14) was observed in patients with relapse who had no prior induction regimen since the current relapse, and the ORR rate was 69.2% (9/13) for patients treated with one prior regimen (**Supplementary Table 3**).

For patients with prior treatment of only the “3+7” induction regimen without any epigenetic modifiers and VEN, the CR rate was 100%, compared to 50% of CRc rate (CR: 41.7%; CRi: 8.3%) for patients who had previously received VEN+HMA regimens (**Supplementary Table 4**). Overall, 35.3% (12 of 34) of the patients had been previously treated with high-dose cytarabine (> 1 g/m^2^ dosing), either as a prior induction therapy or as post-remission therapy. The CRc rate in these patients was 83.3% (10/12; CR: 75%; CRi: 8.3%).

The MRD was evaluated in the entire R/R cohort using multiparametric flow cytometry. Of the 25 patients who achieved a CRc, 11 (44%) also achieved negative MRD at the end of the first induction therapy. There was no difference in MRD negativity rate between patients achieving CR and patients achieving CRi (40.9% *vs*. 66.7%, *P* = 0.5648).

### Clinical responses according to cytogenetic and molecular risks

The CRc rate was 100% (95% CI: 54.1–100) in patients with ELN-favorable risk, 70% (95% CI: 34.8–93.3) in the intermediate risk group, and 66.7% (95% CI: 41.0–86.7) in the adverse risk group (*P* = 0.463) (**Supplementary Table 5**).

**Figure 3:**
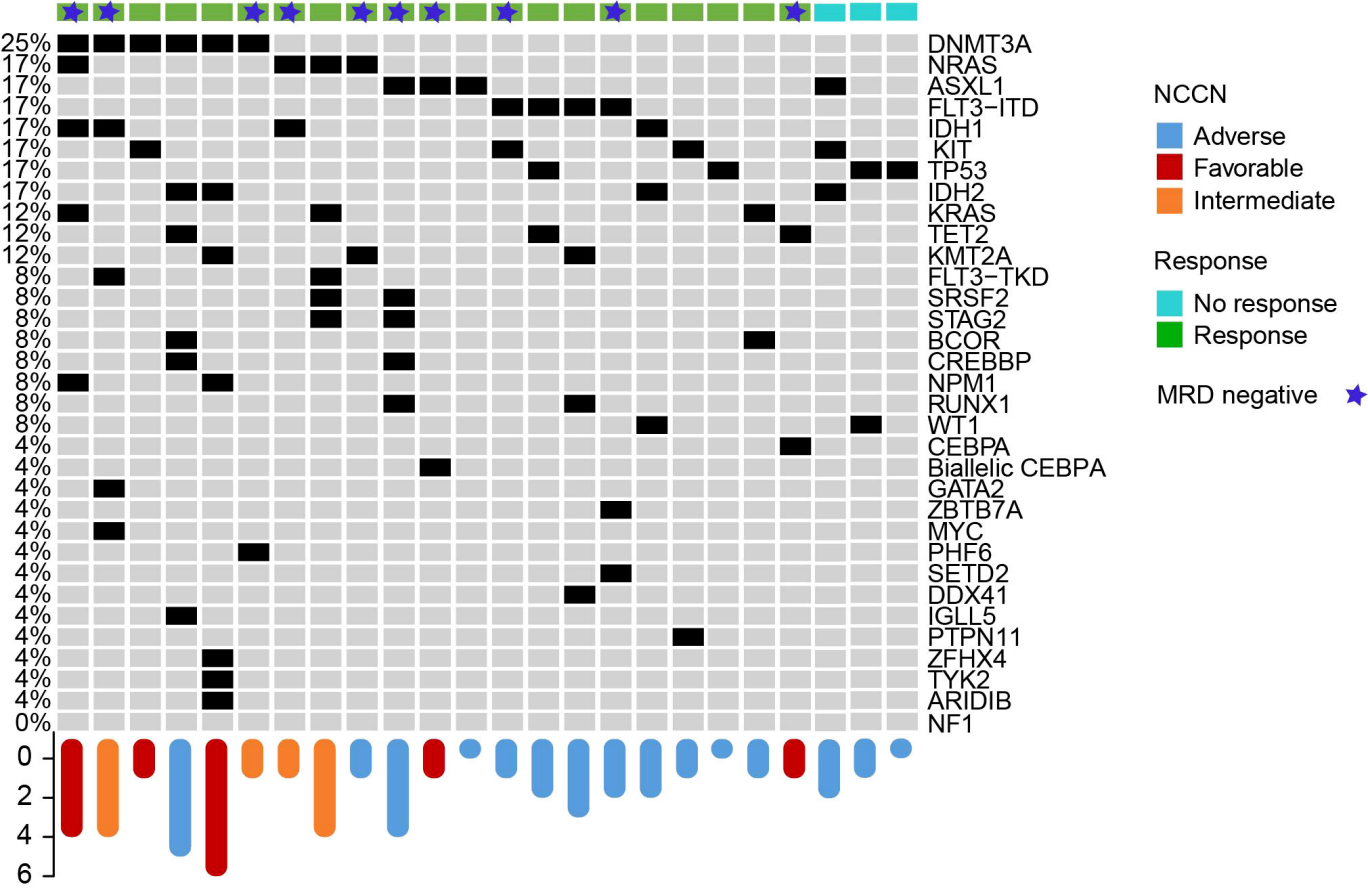
Correlation between somatic mutations and clinical responses.Landscape of mutations detected in 24 patients at enrollment. Each row represents a gene, and each column corresponds to a participant. Bar plots indicate the number of mutations per patient (top bar plot).

To analyze correlations between mutations and responses, we sequenced a 435-gene panel and identified relevant mutations in 34 patients. TP53 mutations were detected in 4/34 patients including three refractory patients and one early relapsed patient. Of which the ORR was 50% (CR: 25%; PR: 25%), two refractory patients had no response to the CACAG-VEN regimen and died on day 54 and day 32, the median OS time was 54 days and the median PFS time was 54 days. For patients with ASXL1 mutations, the ORR was 75% (**Figure 3**).

### Survival

**Figure 4:**
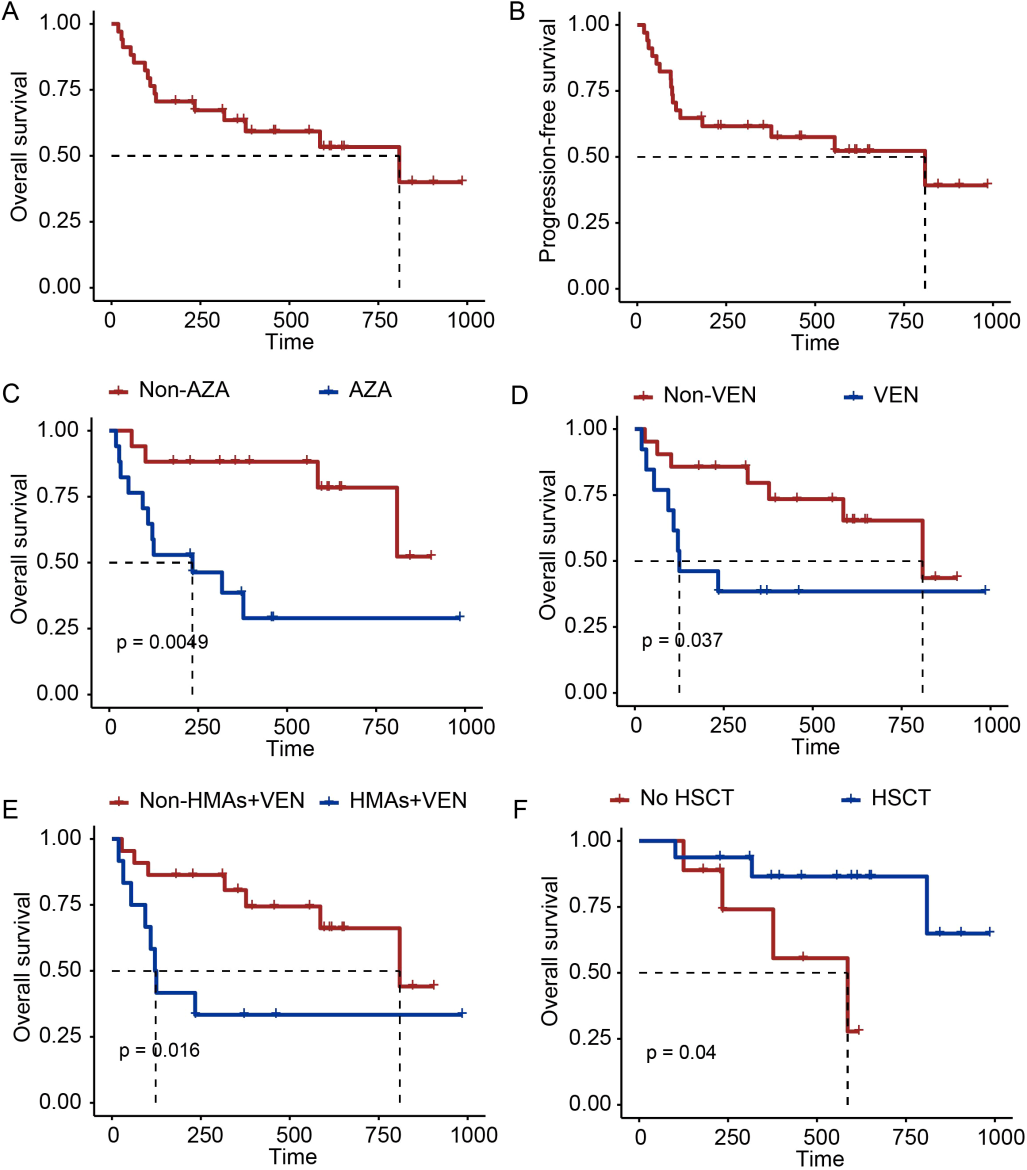
Survival analysis. **(A–B)** Cumulative incidence of overall survival and progression-free survival. **(C–E)** Cumulative incidence of overall survival in patients who had received AZA, VEN **(D)**, HMAs + VEN **(E)**, or not. **(F)** Cumulative incidence of overall survival among patients who did or did not receive allo-HSCT.

As of June 8, 2024, the median follow-up time was 461 days (range: 180–985 days). In the enrolled patients, the 6-month and estimated 1-year OS rates were 70.6% (95% CI: 56.8–87.7%) and 63.5% (95% CI: 48.8–82.5%), respectively (**Figure 4A**). The 6-month and estimated 1-year PFS rates were 64.7% (95% CI: 50.5–82.9%) and 61.6% (95% CI: 47.2–80.4%), respectively (**Figure 4B**). At the end of the follow-up, 15 patients (44.1%) had died. The most common causes of death were relapse (n = 5)/non-remission (n = 7) and infections (pneumonia [n = 1], infection shock [n = 1]).

For patients who achieved a CRc, the 6-month and estimated 1-year OS rates were 92.0% (95% CI: 82.0–100%) and 82.3% (95% CI: 67.8–99.9%); the 6-month and estimated 1-year PFS rates were 84.0% (95% CI: 70.8–99.7%) and 79.8% (95% CI: 65.4–97.3%,**Supplementary Figure 1A**); and the 6-month and 1-year DORs were 84.6% (95% CI: 71.8–99.7%) and 74.8% (95% CI: 59.0–94.9%). Moreover, among these patients who achieved a CRc, MRD-negative patients showed a trend of increased OS and PFS compared to MRD-positive patients, albeit with non-significant statistical differences between the groups (6-month OS, MRD– vs. MRD+: 90.9% *vs*. 92.9%, *P* = 0.58; 6-month PFS: 83.3% *vs*. 60.0%, *P* = 0.69; **Supplementary Figure 1B**).

Six patients (24%) relapsed among the 25 patients who achieved a CRc, with a median time of 140.5 days (range: 42–555), and the estimated 1-year cumulative incidence of relapse was 20.2% (95% CI: 7.3–37.5%).

We also analyzed the survival status among patients who were exposed to different therapy regimens, and the results showed that prior use of AZA, VEN, and HMAs+VEN significantly lowered patients’ 1-year OS rate (AZA *vs*. Non-AZA: 38.6 *vs*. 88.2, *P* = 0.0049; VEN *vs*. Non-VEN: 38.5 *vs*. 79.6, *P* = 0.037; HMAs+VEN *vs*. Non-HMAs+VEN: 33.3 *vs*. 80.6, *P* = 0.016; **Figures 4C–E**). Prior use of chidamide demonstrated no significant survival impact. Furthermore, despite decitabine being a hypomethylating agent (HMA), its prior administration was not associated with statistically significant survival difference in this cohort (**Supplementary Figure 1C**).

By the end of follow-up, 18 of the 34 patients (52.9%) underwent allo-HSCT, including 16 patients (88.9%) who achieved a CRc, one (5.6%) who achieved a PR, and one (5.6%) who still had NR. The CRc rate for those patients who received allo-HSCT tended to be higher than that for those who did not, although the difference was not statistically significant (88.9% *vs*. 56.3%, *P* = 0.0523). Of the patients who achieved a CRc, the estimated 1-year OS was 86.5% (95% CI: 70.7–100%) *vs*. 74.1% (95% CI: 16.1–48.4%) for patients who did or did not receive allo-HSCT (*P* = 0.04, **Figure 4F**); and the estimated 1-year cumulative incidence of relapse was 18.8% (95% CI: 4.3–41%) and 23.8% (95% CI: 2.8–56.1%) for patients who did or did not receive allo-HSCT (*P* = 0.332).

### Longitudinal single-cell analysis of pre- and post-treatment patients revealed transcriptional heterogeneity and distinct resistance pathways

**Figure 5:**
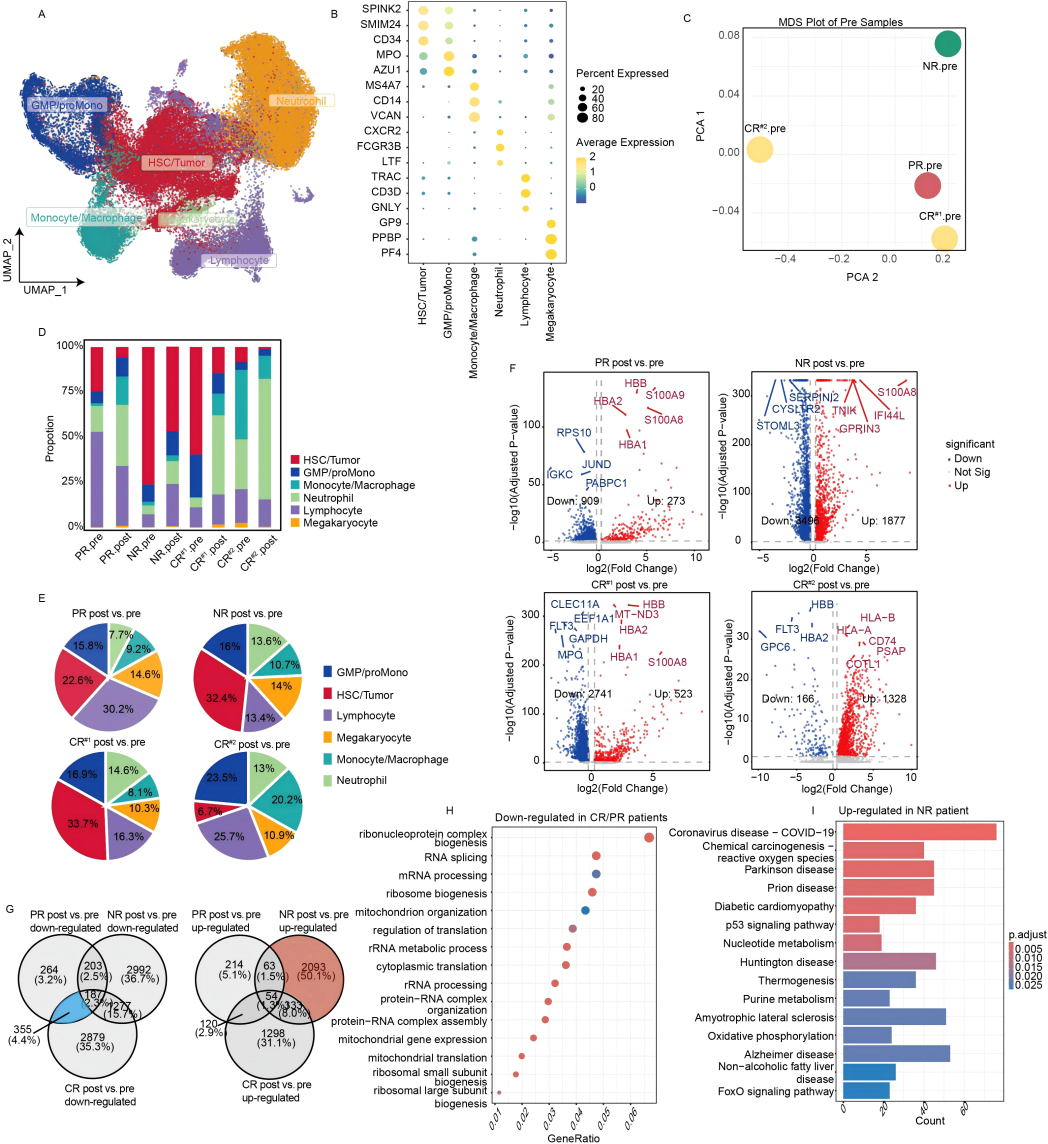
Single-cell transcriptional landscape of patients pre- and post-CACAG-VEN treatment. **(A)** Six major single-cell clusters from BM samples of pre-treatment, and post-treatment patients with R/R AML. Annotation of cell clusters is marked in the bottom part with color codes. **(B)** Dot plot shows the marker gene expression of the identified six major single-cell clusters. Color represents the maximum-normalized mean expression of cells expressing marker genes, and size represents the percentage of cells expressing these genes. **(C)** PCA analysis clustering 4 samples from pre-treatment patients. **(D)** Bar plots show the cellular composition from eight samples derived from four AML patients before and after treatment. **(E)** Pie charts show the distribution of differentially expressed genes (DEGs) from each patient (pre- and post-treatment) across the distinct cell populations. **(F)** volcano plot presents the differential expression analysis comparing pre- versus post-treatment tumor cells for each individual patient. **(G)** Venn diagram (left) compares the sets of downregulated genes (post- versus pre-treatment) across patient groups stratified by treatment response. Venn diagram (right) compares the sets of upregulated genes (post- versus pre-treatment) across patient groups stratified by treatment response. **(H)** GO enrichment analysis of down-regulated genes (post- versus pre-treatment) specifically in responders. **(I)** KEGG enrichment analysis of up-regulated genes (post- versus pre-treatment) specifically in non-responders.

To comprehensively investigate the transcriptional dynamics throughout treatment, single-cell sequencing was performed on pre- and post-treatment samples from four patients. We analyzed 52309 bone marrow mononuclear cells (BMMCs) derived from pre- and post-treatment samples, all of which passed stringent quality controls (**Supplementary Table 6**). After integration of all samples for cluster analysis, we identified six major cell subsets in the BM (**Figure 5A**), in which we observed distinct separation of HSCs/tumor cells with notable enrichment of CD34+ (**Figure 5B**). PCA revealed a dispersed distribution of samples predominantly according to individual patient-specific biological variation rather than clinical response categories, suggesting that baseline transcriptional variation is not the primary driver of treatment outcome (**Figure 5C**; **Supplementary Figure 2A**). Following treatment, we observed a marked reduction in tumor cell proportions copy number variation (CNV) clones, consistent with the intended therapeutic effect (**Figure 5D**; **Supplementary Figures 2B, C**).

We next performed differential gene expression analysis between post- *vs*. pre-treatment samples for each of the four patients. Across the eight sample comparisons, we identified a total of 11,304 differentially expressed genes, the majority of which were downregulated post-treatment (**Supplementary Figure 2D**). Cell-type-specific localization of these differential expression genes indicated that the most substantial transcriptional changes occurred within the tumor cell compartment (**Figures 5E, F**).

To further explore genes associated with treatment response, we focused on differential expression genes within the tumor cell subpopulation for each patient. Notably, we observed downregulation of MCL1, HIF1A, and ABCC1 expression in post-treatment samples (**Supplementary Figure 2E**). By overlapping the sets of downregulated genes (post- versus pre-treatment) specifically in responders, we identified 355 genes that were consistently decreased in patients who responded to CACAG-VEN but remained unchanged in the non-responder (**Figure 5G**). GO enrichment analysis linked these genes to multiple mitochondrial-related processes, suggesting that successful treatment may involve the suppression of mitochondrial activity in tumor cells (**Figure 5H**). Conversely, we detected 2,093 genes that were upregulated (post- versus pre-treatment) in the non-responder post-treatment but not in responders (**Figure 5G**). KEGG analysis demonstrated that these up-regulated (post- versus pre-treatment) genes were enriched in the p53 pathway in this non-responding patient, suggesting activation of p53 signaling pathway in non-responding tumor cells (**Figure 5I**).

## Discussion

This phase 1 single-arm study provides evidence that the addition of VEN, azacitidine and chidamide to the CAG regimen has promising efficacy in R/R AML after one cycle of therapy. Furthermore, the CACAG-VEN regimen was well-tolerated, with a rapid recovery of myelosuppression, with a median time to recovery of 17 days for neutrophil and 24 days for platelets following induction therapy.

The CACAG-VEN regimen showed promising response rates, with an ORR of 76.5% and a composite CRc rate of 73.5% after one cycle of CACAG-VEN, followed by a MRD-negative rate of 44% in patients who achieved CRc. A previous study reported that the efficacy of different VEN-based chemotherapy regimens in patients with R/R AML varies widely, with CR rates ranging from 20% to 67% (29–32). Reports have shown an ORR of 19–29% when using VEN as a monotherapy at 800 mg/d in R/R AML (29, 33). In contrast, VEN in combination with HMA or LDAC increased the ORR from 20.7% to 38.7% (13, 15, 29, 34). In the present study, we observed a higher response rate compared to other VEN-based studies following a single cycle of CACAG-VEN treatment. Furthermore, benefits were observed across all analyzed subgroups. Patients with adverse-risk features, showed a high response rate of 72.2% with CACAG-VEN treatment. The influence of prior therapies on patient responses is a critical consideration in the R/R AML setting. Of all the enrolled patients, 20 (58.8%) had previously failed “3+7” therapy, indicating a chemoresistant leukemic cell population, of which the ORR was 80%. Noted to be in a setting of patients with prior treatment of AZA, VEN, and HMAs+VEN, the ORR rates can still reach 58.8%, 58.3%, and 50%, respectively. The heterogeneous treatment history of our cohort, including exposure to HMAs, conventional chemotherapy, or VEN, likely shaped the subsequent sensitivity to the CACAG-VEN regimen. The synergistic design of our combination is precisely intended to overcome common resistance pathways acquired from these prior treatments. For instance, resistance to VEN monotherapy is frequently mediated by compensatory upregulation of alternative anti-apoptotic proteins like MCL1. Our regimen counteracts this by incorporating aclarubicin and azacitidine, which have been shown to suppress MCL1 expression, thereby resensitizing leukemic cells to BCL2 inhibition. Similarly, the use of an HMA and the HDAC inhibitor chidamide may reverse epigenetic changes that confer resistance to prior cytotoxic therapies. Consequently, the observed efficacy in a significant proportion of pretreated patients can be interpreted as a successful override of pre-existing or acquired resistance mechanisms through multi-targeted inhibition. Interestingly, we found that in patients who had previously used the “3+7” regimen without any epigenetic modifiers and VEN, the CRc rate could reach 100%. These results underscore the potential of the CACAG-VEN combination in consolidating therapeutic efficacy and offer valuable insights for future treatment strategies.

The results of survival analyses suggest that the response to CACAG-VEN significantly improved the survival rate of patients. Compared to patients who had no prior use of AZA, VEN, or HMAs+VEN, the OS was significantly decreased in patients who had prior use of these drugs. Given that we consider that this regimen may be useful in patients with R/R AML who have failed induction with only the “3+7” regimen in the past, it should be used early as a salvage induction regimen instead of the HMAs+VEN regimen or “3+7” regimen to minimize the risk of the patient’s VEN/HMAs/VEN+HMA resistance to the point of failure of induction.

With the previous CDCAG regimen, we observed that patients with FLT3-ITDs had a lower ORR (29.4%), while the addition of VEN improved this. The CRc rate with CACAG-VEN in patients with FLT3-ITDs was 100%. In addition, some studies have suggested that the sensitivity to VEN-based therapy is related to molecular mutations of AML (35, 36), in that patients with FLT3^mut^, TP53^mut^, K/NRAS^mut^, SF3B1^mut^, or DNMT3A^mut^ experienced poor responses (15, 35–38). However, in our clinical trial, we observed poor response rates (50%) mainly in patients carrying TP53. Of the three patients with detectable mutations who did not respond to our new regimen, one patient carried the TP53 and WT1 mutations, one patient carried the TP53 mutation, and one patient carried the AXSL1, KIT, and IDH2 mutations. The addition of epigenetic modifiers may have improved resistance to VEN-based therapy in patients with other mutations, but we need more options when it comes to TP53, and the combination of TP-53-targeting drugs may be the next step.

Single-cell transcriptomic analysis provided further mechanistic insights into treatment response. We found that the transcriptional differences between responders and non-responders were primarily driven by changes within the tumor cell compartment. Notably, we observed downregulation of MCL1, HIF1A, and ABCC1 expression in post-treatment samples, demonstrating the multi-targeting capability of the CACAG-VEN combination in simultaneously addressing key resistance pathways. The downregulation of genes involved in mitochondrial-related processes specifically in responders suggest that successful treatment with the CACAG-VEN regimen involves a coordinated suppression of cellular energy metabolism and stress-response signaling in tumor cells. Conversely, the upregulation of genes related to oxidative phosphorylation, and p53 signaling in the non-responder indicates that activated mitochondrial energy metabolism and p53 pathway may represent key mechanisms of resistance. This aligns with the growing recognition that metabolic reprogramming, particularly dependencies on oxidative phosphorylation (OXPHOS) and mitochondrial function, is a hallmark of AML and a key contributor to therapy resistance (39–42). For instance, targeting OXPHOS with agents like metformin has been shown to induce ferroptosis in AML, with stronger effects observed in specific genetic subtypes such as those with IDH2 and FLT3 mutations. Furthermore, recent research highlighted that reduced mitochondrial apoptotic priming is a fundamental mechanism underlying acquired multi-drug resistance in relapsed AML, independent of the initial genetic background. Beyond mitochondrial dynamics, metabolic reprogramming also plays a crucial role in maintaining the chemoresistance and immune evasion of leukemia stem cells (LSCs). Recent research demonstrated that inhibiting the phosphatase SHP-1 can target resistant LSCs by promoting metabolic reprogramming, thereby enhancing chemosensitivity and restoring immune surveillance (43).

We also demonstrate the tolerability and safety of the combination therapy. The main concerns when combining the six agents was the potential for increased side effects, particularly myelosuppression and infections. VEN was administered on days 1–28 of the VEN-based two-drug combination regimen for R/R AML (44, 45). VEN was administered for 7–14 days per course in the VEN-based three- or four-drug combination regimen for high-risk myelodysplastic syndrome or R/R AML (22, 46). To acknowledge this, with reference to other clinical trial protocols, we implemented a reduction in VEN dosing from 28 to 14 days per course (23). The goal was to maximize the potentiation of VEN during the course of combination chemotherapy and allow sufficient time for marrow recovery. The results of the CACAG-VEN regimen were encouraging, and the regimen was well-tolerated with low treatment-related mortality. This was reflected by the single early death due to infectious shock, which was rigorously assessed and concluded to be a consequence of the patient's underlying immunocompromised state from R/R AML, rather than the study regimen. Compared to VEN in combination with HMAs treatment, 51 of 71 (71.8%) patients remained neutropenic for ≥ 30 days during treatment. In this trial, the CACAG-VEN regimen did not seem to delay count recovery or increase the incidence of serious AEs that would have been expected with VEN in combination with HMA treatment. Importantly, early mortality rates and severe comorbidities (e.g., bacteremia and pneumonia) were not increased compared to those reported when HMAs and VEN are used alone (32).

Despite these promising observations, our study is limited by a relatively small, single-arm Phase 1 cohort. Therefore this study has several limitations, particularly concerning the assessment of drug safety. A more comprehensive and definitive evaluation of safety will require further investigation in larger, randomized controlled trials.

## Conclusions

Generally, CACAG-VEN represents an effective reinduction regimen for R/R-AML, with particular utility as a frontline salvage regimen in R/R patients who only failed the “3+7” regimen. Confirmation of a longer follow-up period with a greater durability of responses and long-term survival is necessary to confirm the safety and effectiveness.

## Data Availability

The original datasets generated in this study have been deposited in the NCBI Gene Expression Omnibus (GEO) repository under accession number GSE311458 and are available at: https://www.ncbi.nlm.nih.gov/geo.

## Glossary

AML: Acute myeloid leukemia
R/R-AML: Relapsed or refractory AML
CACAG-VEN: Chidamide, cytarabine, granulocyte colony-stimulating factor, and aclarubicin in combination with the B cell lymphoma-2 inhibitor venetoclax and azacitidine regimen.
ORR: Overall response rate
NGS: Next-generation sequencing
OS: Overall survival (OS)
PFS: Progression-free survival rate (PFS)
DOR: Duration of response
HSCT: Hematopoietic stem cell transplantation (HSCT)
BCL2: B-cell leukemia/lymphoma-2 (BCL2)
MCL1: Myeloid cell leukemia 1 (MCL1)
BCL-XL: B cell lymphoma extra-large (BCL-XL)
VEN: Venetoclax
AZA: Azacitidine
DAC: Decitabine
HMAs: Hypomethylating agents (HMAs)
HDAC: histone deacetylase
G-CSF: Granulocyte colony-stimulating factor (G-CSF)
CAG: Cytarabine, aclarubicin and G-CSF regimen
CDCAG: CAG regimen along with chidamide and decitabine
CI: Confidence interval
CR: Complete response
PR: Partial response
NR: No response
MRD: Measurable residual disease
QRT-PCR: Real-time quantitative polymerase chain reaction
ELN: European LeukemiaNet
BM: Bone marrow
PB: peripheral blood
AE: Adverse events
NCI: National Cancer Institute
BMMCs: Bone marrow mononuclear cells
HCs: Healthy controls
CNV: Copy number variation
HSCs/GMPs: Hematopoietic stem cells and granulocyte-macrophage progenitors
DEGs: Differentially expressed genes
GO: Gene ontology
KEGG: Kyoto Encyclopedia of Genes and Genomes
