# Supplementary material for: Phase 1 study of chidamide in combination with venetoclax, azacitidine, aclarubicin, cytarabine and G-CSF for refractory/relapsed acute myeloid leukemia: clinical safety, efficacy, and correlative analysis
Source: Front Immunol. 2025 Dec 11;16:1698710. doi: 10.3389/fimmu.2025.1698710 (PMC12738368; doi:10.3389/fimmu.2025.1698710)
Supplement: Supplementary file 1 [file DataSheet1.pdf]

CLINICAL STUDY PROTOCOL

Protocol Title: Single arm, open label, phase 1 clinical study of venetoclax combined with CACAG regimen in the treatment of relapsed/refractory acute myeloid leukemia

Protocol number: ChiCTR2200065634
Study regimens: CACAG+VEN

Study Phase: 1

Indication: Salvage therapy of relapsed/refractory acute myeloid leukemia
Application Institution: Chinese PLA General Hospital
Principal Institution: Chinese PLA General Hospital

INVESTIGATOR'S STATEMENT

I have received and completely reviewed the following protocol (ChiCTR2200065634), including all appendices:

As Principle Investigator, I understand and agree to conduct this clinical study as described and will comply with the ethical and regulatory considerations delineated herein.

Study Title

Single arm, open label, phase 1 clinical study of venetoclax combined with CACAG regimen in the treatment of relapsed/refractory acute myeloid leukemia

Principle Investigator Signature and Contact Information

Principle Investigator (print) ______________________________

Principle Investigator (signature) ______________________________

Date of Signature ______________________________

Institution/Affiliation ______________________________

City, Province, Country ______________________________

Study Synopsis

**Study title**	Single arm, open label, phase 1 clinical study of venetoclax combined with CACAG regimen in the treatment of relapsed/refractory acute myeloid leukemia
**Protocol number**	ChiCTR2200065634
**Indication**	Salvage therapy of relapsed/refractory acute myeloid leukemia
**Study phase**	1
**Study applicant**	Chinese PLA General Hospital
**Study centers**	Chinese PLA General Hospital
**Number of subjects planned**	34
**Study duration**	Estimated to be 2 years
**Objectives**	
**Primary objective**	The primary endpoint was the treatment-related adverse events and ORR after one cycle of CACAG+VEN treatment.
**Secondary objectives**	The secondary endpoints included CRc [complete response (CR) plus complete response with incomplete blood count recovery (CRi)] rate, partial response (PR) rate, no response (NR) rate, MRD negative rate after one cycle, OS, progression-free survival (PFS), duration of response (DOR) and cumulative incidence of relapse

**Study design**	All patients in this study were treated with the CACAG+VEN regimen over a 28-day cycle: VEN was administered orally (PO) on days 1–14 (100 mg for day 1, 200 mg for day 2, 400 mg for days 3–14); chidamide 30 mg was administered orally on days 1, 4, 8, and 11; AZA 75 mg/m^2 was administered subcutaneously on days 1–7; cytarabine 75–100 $mg/m^2/d$ was administered on days 1–5, intravenously; aclarubicin 20 mg/d was administered on days 1, 3, and 5; and granulocyte colony-stimulating factor (G-CSF) 300 μg/day was administered until WBC > 20 × 10^9/L. BM assessment for response was performed on day 28 after starting treatment. Posaconazole was administered as a preventive measure against fungal infections. If patients required antifungal treatment or other moderate or strong CYP3A4 inhibitors, the venetoclax dose was adjusted according to the prescription information recommendations. Supportive treatment, including anti-infection prophylaxis and growth factor support, was permitted at the investigator's discretion. Morphologic, cytogenetic, and MRD assessments were performed during each BM assessment.
**Inclusion criteria**	Voluntary participation in the clinical study; the patient or their legal guardian fully understands and is informed of the study and signs the informed consent form (ICF); is willing to follow and complete all of the trial procedures.<br>Patients aged 14–75 years with refractory or relapsed (R/R) AML (not acute promyelocytic leukemia), diagnosed in accordance with the 2021 edition of the CMA criteria.<br>Liver function: serum aspartate aminotransferase or alanine aminotransferase ≤ 2.5 × upper limit of normal range (ULN), serum bilirubin ≤ 2 × ULN.<br>Kidney function: serum creatinine ≤ ULN.

	No uncontrollable infections or serious psychiatric disorders.<br>Eligible patients were required to have an ECOG performance status ≤ 2.
**Exclusion criteria**	Pregnant or lactating.<br>Patients with chronic alcohol abuse that affects evaluation of the test results.<br>Patients with mental illness or other conditions that prevent informed consent to complete the treatment and examination steps.<br>Patients less than 6 weeks after surgery on a vital organs of the body.
**Study treatment**	VEN was administered orally (PO) on days 1–14 (100 mg for day 1, 200 mg for day 2, 400 mg for days 3–14); chidamide 30 mg was administered orally on days 1, 4, 8, and 11; AZA 75 mg/m^2 was administered subcutaneously on days 1–7; cytarabine 75–100 mg/m^2/d was administered on days 1–5, intravenously; aclarubicin 20 mg/d was administered on days 1, 3, and 5; and granulocyte colony-stimulating factor (G-CSF) 300 μg/day was administered until WBC > 20 × 10^9/L. BM assessment for response was performed on day 28 after starting treatment. Posaconazole was administered as a preventive measure against fungal infections. If patients required antifungal treatment or other moderate or strong CYP3A4 inhibitors, the venetoclax dose was adjusted according to the prescription information recommendations.
**Sample size determination**	The sample size was calculated according to the primary endpoint (ORR) of the study. Our previous study reported that the ORR of patients with R/R AML treated with the CDCAG regimen was 46.2%. Additionally, several clinical trials revealed that the ORRs for adult patients with R/R AML receiving venetoclax along with intensive chemotherapy (FLAG-IDA) were 72–75%. Therefore, an

	expected ORR of 75% was established for patients treated with the CACAG+VEN regimen. A sample size of 27 achieves 90% power to detect differences using a two-sided exact test with a significance level (alpha) of 0.05 (PASS software, NCSS LCC, USA). Allowing a drop-out rate of 20%, 34 patients were required.
**Statistical analysis**	Statistical analysis is performed based on the intent to treat (ITT) population, which includes all subjects. The patient status was determined through telephone follow-ups and the review of outpatient or inpatient records. Continuous data are described as the median with range or mean and standard deviation (SD) according to the normality of the distribution. Categorical data are described as n (%). The ORR, CR, CRi, CRc, and MRD-negative rates were calculated using 95% confidence interval (CI). The Kaplan–Meier method was used to estimate the DOR, PFS, and OS. The cumulative incidence of relapse was estimated using a competing risk model. Death without relapse was defined as a competing event for relapse. Safety analysis was used to calculate the frequency of various events and the ratio of the severity of each event by detailing the cases of blood toxic or nonblood toxic reactions. A two-sided P value < 0.05 was considered to indicate statistical significance. All statistical analyses were performed using Easy R (version 1.61) and R (version 4.3.3).

TABLE OF CONTENTS

CLINICAL STUDY PROTOCOL 1

INVESTIGATOR'S STATEMENT 2

Study Synopsis 3

TABLE OF CONTENTS 7

Abbreviations 9

1.Introduction 11

2. Study Design 12

3. Subject Selection Criteria 14

- 3.1 Subject Selection Criteria 14
  - 3.1.1 Number of subjects 14
  - 3.1.2 Inclusion Criteria 15
  - 3.1.3 Exclusion Criteria 15
- 3.2 Withdraw Criteria 15

4. Study Procedures 16

- 4.1 Screening 16
- 4.2 Study Treatment 16
- 4.3 Follow-up 17

5. Efficacy Assessments 18

- 5.1 Definitions 18
- 5.2 Primary Efficacy Endpoint 19
- 5.3 Secondary Efficacy Endpoints 19
- 5.4 Schedule and methods of Efficacy Assessments 19

6. Safety Evaluation 19

- 6.1 Medical History 20
- 6.2 Vital Signs and Physics Examination 20
- 6.3 Clinical Symptoms 20
- 6.4 Clinical Laboratory Evaluations 21

7. Adverse Events and Serious Adverse Events (SAEs) 21
7.1 Definitions 21
7.1.1 Adverse Events 21
7.1.2 Serious Adverse Events 22
7.2 Assessment of Severity 22
7.3 Assessment of Causality 22
7.4 Recording and Reporting AEs and SAEs 23
8. Rules of Withdrawal 23
8.1 Subjects withdraw from the Stud 23
8.2 Premature Termination of the Study 24
9. Roles of Follow-Up 24
9.1 Follow-up period 24
9.2 Visit Scheduling 24
9.3 Contents 25
10. Data Analysis and Statistical Considerations 25
10.1 Sample Size Assumptions 25
10.2 Primary Efficacy Endpoint 25
10.3 Secondary Efficacy Endpoints 25
10.4 Data Analysis Considerations 26
10.4.1 Analysis Population 26
10.4.2 Analysis Plan 26
11. Materials for the Study 27
12. Ethical Considerations 27
12.1 Responsibilities of Investigators 27
12.2 Informed Consent Process 27
12.3 Good Clinical Practice 28
12.4 Protection of Subjects' Personal Data 28
13. Administrative Requirements 28
14. References 29

Abbreviations

R/R	Relapsed/refractory
AML	Acute myeloid leukemia
ORR	Overall response rate
CR	Complete response
CRi	Complete response with incomplete hematologic recovery
PR	Partial response
CRc	Complete response (CR) plus complete response with incomplete blood count recovery (CRi)
OS	Overall survival
PFS	Progression-free survival
DOR	Duration of response
CIR	Cumulative incidence of relapse
VEN	Venetoclax
AZA	Azacitidine
G-CSF	Granulocyte colony-stimulating factor
Allo-HSCT	Allogeneic hematopoietic stem-cell transplantation
ECOG	Eastern Cooperative Oncology Group
MRD	Measurable residual disease
ULN	Upper limit of normal
ALT	Alanine aminotransferase
AST	Aspartate aminotransferase
HMAs	Hypomethylating agents
CI	Confidence interval
BCL-2	Anti-apoptotic B-cell lymphoma 2
BCL-XL	B cell lymphoma extra-large

MCL-1	Myeloid cell leukemia 1
HDAC3	Histone deacetylase 3
AKT	Serine-threonine kinase
CDK2	Cyclin-dependent kinase 2
DAC	Decitabine
LDAC	Low-dose cytarabine
CAG	Cytarabine, aclarubicin and granulocyte-colony-stimulating factor
VA	Venetoclax and azacitidine
CDCAG	Chidamide, decitabine, cytarabine, aclarubicin and granulocyte colony-stimulating factor
BM	Bone marrow
PB	Peripheral blood
AEs	Adverse events
CTCAE	Common Terminology Criteria for Adverse Events
ITT	Intent-to-treat
CRF	Case report form
GCP	Chinese good clinical practice

1.Introduction

Acute myeloid leukemia (AML) is a heterogeneous and aggressive hematopoietic malignancy. Despite great advances in targeted therapy, chemotherapy, and hematopoietic stem cell transplantation (HSCT), up to 35–45% of patients are refractory to conventional 3+7 intensive therapy or relapse [1-4]. The prognosis of refractory/relapsed (R/R) AML is dismal, with a median overall survival (OS) of 3–7 months [5]. Currently, there is no standard salvage therapy for R/R AML, indicating an urgent need for novel treatment to improve the outcomes [5-8].

B-cell leukemia/lymphoma-2 (BCL2) is an antiapoptotic protein commonly expressed in hematologic malignancies, playing a role in tumor survival and chemoresistance [9-11]. Resistance to venetoclax is mediated by other pro-survival proteins, such as myeloid cell leukemia 1 (MCL1) and B cell lymphoma extra-large (BCL-XL) [12]. Hypomethylating agents (HMAs) might synergistically inhibit MCL1 and BCL-XL, thereby increasing the dependence of leukemia cells on BCL-2 [12]. Recent studies have shown that a low-intensity regimen combining venetoclax (VEN) with HMAs such as azacitidine (AZA), decitabine (DAC), or low-dose cytarabine (LDAC), improves response and survival rates in patients with R/R AML [13-15]. Aldoss et al. assessed the efficacy of combining VEN and HMAs in 90 patients with R/R AML, achieving a response rate of 46% [16-17]. However, 30–45% of patients with R/R AML or who are unfit and have AML still fail to achieve complete remission with a low-intensity regimen [18]. It is important to explore effective and safe treatment options.

A previous study showed that combining HMAs with the CAG regimen was well tolerated and improved prognosis in elderly patients with high-risk AML [19-20]. We previously used the CAG regimen along with chidamide and DAC (CDCAG) for R/R AML. The CDCAG regimen was well tolerated and showed moderate anti-leukemic activity with a complete response rate or complete response with incomplete blood count recovery (CR/CRi) rate of 46.2%, overall response rate (ORR) of 54.8%, median overall survival (OS) of 266 days, and 1-year OS rate of

36.9%. In accordance with the clinical trial results, our preclinical data showed that chidamide can increase the sensitivity to anthracyclines via regulation of the HDAC3-AKT-P21-CDK2 signaling pathway in R/R AML cells [21]. These results are encouraging, indicating that epigenetic modifiers combined with cytotoxic agents may represent a promising treatment direction for patients with R/R AML [22].

These data provide a strong clinical rationale for the combination of chidamide with the VA and CAG regimen (CACAG+VEN) for the treatment of R/R AML. Therefore, we conducted a single-center phase 1 trial to investigate the efficacy and tolerability of the CACAG+VEN regimen for patients with R/R AML.

2. Study Design

This is a single-arm, phase 1 clinical study to investigate the efficacy and tolerability of CACAG+VEN regimen in patients with R/R AML. Approximately 34 subjects will be enrolled to receive CACAG+VEN regimen for salvage therapy. The study design is illustrated in Figure 1.

Subjects with R/R AML will be screened for eligibility. Medical history evaluation, vital sign, physical examination, ECOG performance status, blood and urine sampling for laboratory tests, electrocardiogram, chest imaging examination as well as bone marrow (BM) assessment will be performed to determine study eligibility.

After enrolment, patients will be assigned to the trial therapy. All patients in this study were treated with the CACAG+VEN regimen over a 28-day cycle: VEN was administered orally (PO) on days 1–14 (100 mg for day 1, 200 mg for day 2, 400 mg for days 3–14); chidamide 30 mg was administered orally on days 1, 4, 8, and 11; AZA 75 mg/m^2 was administered subcutaneously on days 1–7; cytarabine 75–100 $mg/m^2/d$ was administered on days 1–5, intravenously; aclarubicin 20 mg/d was administered on days 1, 3, and 5; and granulocyte colony-stimulating factor (G-CSF) 300 μg/day was administered until WBC > $20 \times 10^9/L$. BM assessment for response was performed on day 28 after starting treatment. Posaconazole was administered as

a preventive measure against fungal infections. If patients required antifungal treatment or other moderate or strong CYP3A4 inhibitors, the venetoclax dose was adjusted according to the prescription information recommendations. Supportive treatment, including anti-infection prophylaxis and growth factor support, was permitted at the investigator's discretion. Morphologic, cytogenetic, and MRD assessments were performed during each BM assessment. Once acquiring CRc, the patients are recommended to receive allo-HSCT if donors are available. If donors are unavailable, patients will receive one course of original therapy again and sequential cytarabine-based consolidation therapy. In addition, for the patients undergoing allo-HSCT, sorafenib maintenance post-transplantation was recommended regardless of FLT3 being mutated or not. If patients do not obtain CRc after one course of the trial therapy, they will proceed to allo-HSCT if donors are available, and patients receive other salvage therapy based on patients' personal preferences after full discussion with physicians if donors are unavailable.

For response assessments, bone marrow is evaluated on cycle 1 day 28 and again 1-2 weeks after hematological recovery if the day 28 bone marrow is aplastic. Morphological and MRD assessments are done during each bone marrow assessment. CR was defined as the disappearance of signs and symptoms of leukemia, absence of leukemic cells in the leukocyte classification, $< 5\%$ primitive cells in the BM, absence of extramedullary leukemia, and neutrophil count $\geq 1.0 \times 10^9$/L, PLT $\geq 100 \times 10^9$/L; a CRi was defined as the disappearance of signs and symptoms of leukemia, absence of leukemic cells in the leukocyte classification, $< 5\%$ primitive cells in the BM, absence of extramedullary leukemia, and neutrophil count $< 1.0 \times 10^9$/L or PLT $< 100 \times 10^9$/L; PR was defined as a decrease of BM primitive cells to 6–20% or decreased over 50% from pretreatment; NR was defined as non-fulfillment of the above criteria; MRD was evaluated with a sensitivity of 0.01–0.10%, defining MRD negative as $< 0.1\%$ and MRD positive as 0.1% or higher. The clinical data cutoff date is June 8, 2024. Criteria for removing patients from trial therapy includes the development of intolerable adverse events related to study

treatment (determined by the treating physician), patient withdrew informed consent, and completion of the protocol therapy and evaluation period.

Figure 1 Study Schema

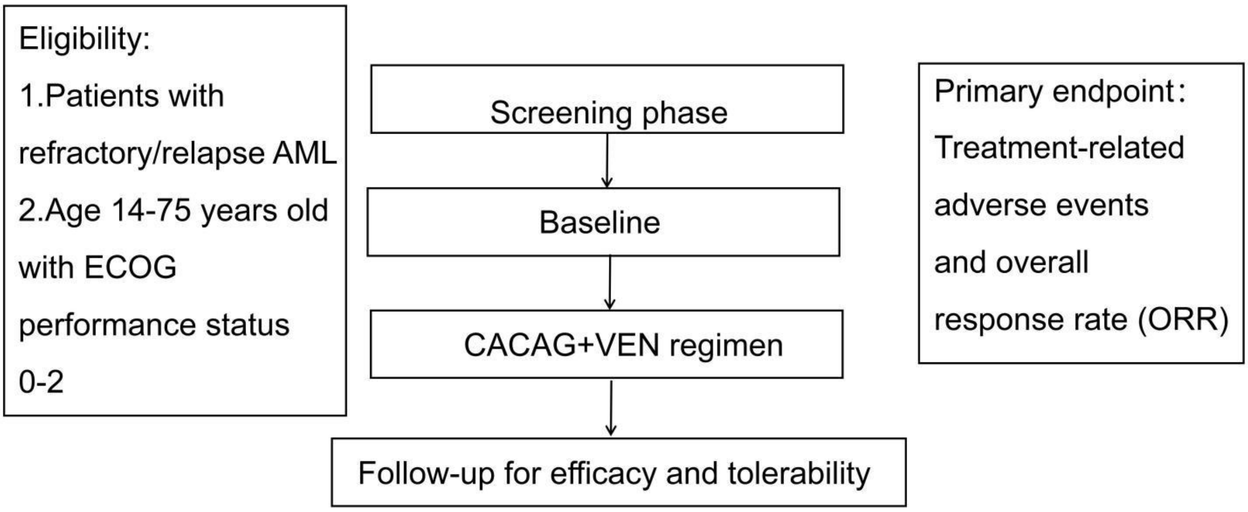

AML: acute myeloid leukemia; ECOG: Eastern Cooperative Oncology Group; ORR: overall response rate; CACAG+VEN: chidamide + azacytidine + cytarabine + aclarubicin + G-CSF + venetoclax

3. Subject Selection Criteria

3.1 Subject Selection Criteria

3.1.1 Number of subjects

Approximately 34 subjects will be enrolled to receive CACAG+VEN regimen for salvage therapy.

3.1.2 Inclusion Criteria

Subjects eligible for enrolment in this study must meet all of the following criteria:

(1) Patients with R/R AML:

Refractory AML was defined as no CRc and a reduction in bone marrow (BM) blast count of 50% after one cycle or no CRc after two cycles [23] [24]. Relapsed AML was defined as recurrence of blasts in the peripheral blood (PB), BM blasts ≥ 5%, or development of extramedullary disease after achieving a CRc.

(2) Age 14 to 75 years old with ECOG performance status of 0-2.

(3) Sign informed consent form, have the ability to comply with study and follow-up procedures.

3.1.3 Exclusion Criteria

Subjects meeting any of the following criteria are ineligible for this study:

(1) Hepatic abnormalities (serum aspartate aminotransferase or alanine aminotransferase ≥2.5 times the upper limit of normal [ULN], serum bilirubin ≥2 times the ULN).

(2) Renal dysfunction (creatinine ≥the upper limit of normal)

(3) Pregnant or lactating.

(4) Patients with chronic alcohol abuse that affects evaluation of the test results.

(5) Patients with mental illness or other conditions that prevent informed consent to complete the treatment and examination steps.

(6) Patients less than 6 weeks after surgery on a vital organs of the body.

(7) Uncontrolled active infection.

3.2 Withdraw Criteria

Subjects are free to withdraw consent and discontinue participation in the study at any time and without prejudice to future treatment. A subject's participation in the study may be discontinued at any time at the investigator's discretion. Justifiable

reasons for a subject to be withdrawn from the study include:

(1) Inability to fully comply with the study protocol

(2) Unacceptable toxicity

(3) Best interest of the subject based upon the investigator's discretion

(4) At the request of the study subject at any time and for any reason

Subjects will be followed up unless the informed consent is withdrawn. The reason for withdrawal from study participation and the date must be documented in the case report form (CRF). The investigator must complete the last visit, including vital signs, physical examination, laboratory tests, disease status and AE assessment, all of which must be documented in the CRF.

4. Study Procedures

4.1 Screening

Subjects with R/R AML will be screened for eligibility by an independent Data Safety Monitoring Board (DSMB). Medical history evaluation, vital sign, physical examination, ECOG performance status, blood and urine sampling for laboratory tests, electrocardiogram, chest imaging examination as well as BM assessment will be performed to determine study eligibility, all of which must be performed ≤3 days prior to randomization.

4.2 Study Treatment

4.2.1 Dose Escalation and Determination

Patients were assigned sequentially to dose-escalation groups according to a 3+3 design, in which groups that received sequentially higher doses were opened after three patients had completed 3 weeks of treatment at the preceding dose without having dose-limiting toxic effects (DLTs). DLTs were defined as the following events occurring within the DLT evaluation period: grade≥4 nonhematologic toxicity; absolute neutrophil count (ANC) < 500/μL (grade 4) or platelets <

25,000/µL (grade 4) for > 14 days off therapy without evidence of leukemia (< 5% blasts) in the BM or blood, or > 42 days from therapy initiation, whichever is longer. The first dose was the fixed-dosed CACAG regimen and 7 days venetoclax (100 mg for day 1, 200 mg for day 2, 400 mg for days 3–7) . Based on the safety data in the first three patients led to the introduction of stepwise intrapatient increases in dose to the fixed-dosed CACAG regimen and 14 days venetoclax (100 mg for day 1, 200 mg for day 2, 400 mg for days 3–14) for the subsequent dose-escalation and expansion cohorts.

4.2.2 CACAG+VEN salvage therapy

VEN was administered orally (PO) on days 1–14 (100 mg for day 1, 200 mg for day 2, 400 mg for days 3–14); chidamide 30 mg was administered orally on days 1, 4, 8, and 11; AZA 75 mg/m^2 was administered subcutaneously on days 1–7; cytarabine 75–100 $mg/m^2/d$ was administered on days 1–5, intravenously; aclarubicin 20 mg/d was administered on days 1, 3, and 5; and granulocyte colony-stimulating factor (G-CSF) 300 µg/day was administered until WBC > 20 × 10^9/L. BM assessment for response was performed on day 28 after starting treatment. Posaconazole was administered as a preventive measure against fungal infections. If patients required antifungal treatment or other moderate or strong CYP3A4 inhibitors, the venetoclax dose was adjusted according to the prescription information recommendations. Supportive treatment, including anti-infection prophylaxis and growth factor support, was permitted at the investigator's discretion.

4.3 Follow-up

The clinical data cutoff date is June 8, 2024. Disease assessment including routine blood and BM assessment will be regularly performed post-salvage therapy. For response assessments, bone marrow is evaluated on cycle 1 day 28 and again 1-2 weeks after hematological recovery if the day 28 bone marrow is aplastic. Subsequent bone marrow evaluations are done before and after cycles 2, and then as clinically needed. Adverse events are recorded up to 4 weeks after the

discontinuation of the trial therapy, and are graded according to the National Cancer Institute Common Terminology Criteria for Adverse Events version 4.03.

5. Efficacy Assessments

5.1 Definitions

(1) Refractory AML was defined as no CRc and a reduction in bone marrow (BM) blast count of 50% after one cycle or no CRc after two cycles.

(2) Relapsed AML was defined as recurrence of blasts in the peripheral blood (PB), BM blasts ≥ 5%, or development of extramedullary disease after achieving a CRc. CR was defined as the disappearance of signs and symptoms of leukemia, absence of leukemic cells in the leukocyte classification, < 5% primitive cells in the BM, absence of extramedullary leukemia, and neutrophil count ≥ 1.0 × 10^9/L, PLT ≥ 100 × 10^9/L.

(3) CRi was defined as the disappearance of signs and symptoms of leukemia, absence of leukemic cells in the leukocyte classification, < 5% primitive cells in the BM, absence of extramedullary leukemia, and neutrophil count < 1.0 × 10^9/L or PLT < 100 × 10^9/L.

(4) PR was defined as a decrease of BM primitive cells to 6–20% and decreased over 50% from pretreatment.

(5) NR was defined as non-fulfillment of the above criteria; MRD was evaluated with a sensitivity of 0.01–0.10%, defining MRD negative as < 0.1% and MRD positive as 0.1% or higher.

(6) Early relapse was defined as the presence of > 5% BM primitive cells or extramedullary infiltrates after achieving a CR within 12 months, whereas late relapse was defined as relapse occurring beyond 12 months.

(7) OS was defined as the time from enrollment into CACAG+VEN therapy until death from any cause or last follow-up.

(8) PFS was defined as the time from enrollment until relapse or death from any cause, whichever occurred first.

(9) ORR was calculated as CRc+PR.

(10) DOR was defined among responders as the duration between the date of response and the date of disease relapse or death from any cause, whichever occurred first.

5.2 Primary Efficacy Endpoint

The primary endpoint was the treatment-related adverse events and ORR after one cycle of CACAG+VEN treatment.

5.3 Secondary Efficacy Endpoints

The secondary endpoints are CRc rate, PR rate, NR rate, MRD negative rate, OS, PFS, DOR and CIR.

5.4 Schedule and methods of Efficacy Assessments

Disease assessment including routine blood and BM assessment will be regularly performed post-salvage therapy. For response assessments, bone marrow is evaluated on cycle 1 day 28 and again 1-2 weeks after hematological recovery if the day 28 bone marrow is aplastic. Adverse events are recorded up to 4 weeks after the discontinuation of the trial therapy, and are graded according to the National Cancer Institute Common Terminology Criteria for Adverse Events version 4.03.

6. Safety Evaluation

Adverse events are recorded up to 4 weeks after the discontinuation of the trial therapy, and are graded according to the National Cancer Institute Common Terminology Criteria for Adverse Events version 4.03. The study adjudication committee (consisting of experts in hematology, infection, pathology, pharmacy, and statistics) judge whether adverse events are treatment-related or non-treatment-related. Serious adverse events are those that resulted in death, disability, or

incapacity, are life-threatening or judged an important medical event, or required hospitalization or prolongation of existing hospitalization.

6.1 Medical History

Each subject's medical history must be obtained at screening. Information on any prior or existing medical conditions will be recorded on the appropriate CRF.

6.2 Vital Signs and Physics Examination

Vital signs and results of physics examination must be documented before enrollment, once a week for the first two months after enrollment. The next 8 items must be performed:

(1) Physical examination

(2) Heart rate

(3) Blood pressure

(4) Body temperature

(5) Rate of respiration

(6) Body weight

(7) ECOG performance status

(8) Signs of infection

6.3 Clinical Symptoms

During the study, the patients' clinical symptoms must be documented. The clinical symptoms may be associated with the administration of any drugs of the CACAG+VEN regimen reported previously, including rash, dermatitis, erythema, flushing, pruritus, dry skin, alopecia, stomatitis, diarrhea, nausea, vomiting, pancreatitis, weight loss, anorexia, fatigue, fever, headache, sensory neuropathy, myalgia, arthralgia, abdominal pain, cardiac ischaemia, dyspnea, infections, bleeding and hypertension.

6.4 Clinical Laboratory Evaluations

Before initiation of the study, the monitor will document the normal range of each test in every involved laboratory. During the study, the next items must be performed:

(1) Routine blood: white cell counts, neutrophil cell counts, hemoglobin, and platelet counts.

(2) Hepatic function: serum aspartate aminotransferase, alanine aminotransferase, serum bilirubin, lactic dehydrogenase, alkaline phosphatase, albumin and total protein.

(3) Renal function: serum creatinine, urea nitrogen and uric acid.

(4) Other biochemical indicators: amylase and lipase.

(5) Electrolytes: sodium, potassium, calcium and magnesium.

(6) Coagulation function: prothrombin time-international normalized ratio, activated partial thromboplastin time and fibrinogen.

(7) Urinalysis: protein, glucose and erythrocyte.

(8) Electrocardiogram.

(9) Chest imaging examination.

7. Adverse Events and Serious Adverse Events (SAEs)

The investigator is responsible for detecting, documenting and reporting events that meet the definition of AE and SAE.

7.1 Definitions

7.1.1 Adverse Events

AE is any untoward medical occurrence in a subject of a clinical investigation, which does not necessarily have a causal relationship to the medicinal product. Therefore, an AE can be any unfavorable and unintended sign, including an abnormal laboratory finding, symptom, or disease (new or exacerbated), whether or

not it is considered to be related to the product. This definition includes any newly occurring event or previous condition that has increased in severity or frequency since the administration of the product. However, relapse or death due to relapse should not be recorded as AEs.

7.1.2 Serious Adverse Events

A serious AE is any untoward medical occurrence that, at any dose:

(1) Results in death;

(2) Is life-threatening;

(3) Requires quires hospitalization or prolongation of existing hospitalization - ie, the AE requires at least a 24-hour inpatient hospitalization or prolongs a hospitalization beyond the expected length of stay.

Hospitalization or prolongation of existing hospitalization for social reasons will not be reported as SAE.

(1) Results in disability/incapacity;

(2) Congenital anomaly/birth defect;

(3) Important medical event.

Medical or scientific judgment should be exercised in deciding whether SAE reporting is appropriate in other situations. An important medical event is an event that may not result in death, be life-threatening, or require hospitalization, but is clearly of major clinical significance. The AE may jeopardize the subject or require intervention to prevent a serious outcome.

7.2 Assessment of Severity

All AEs are graded according to CTCAE version 4.03.

7.3 Assessment of Causality

The investigator must determine the relationship of each AE and SAE to study treatment. Relationship of AE or SAE to study treatment will be defined according

to the following criteria:

(1) Definite: There id a clear temporal relationship to study treatment, with no other possible cause.

(2) Possible: A temporal relationship to study treatment is not clear, and alternative etiologies are possible.

(3) Not related: There is no temporal relationship to study treatment, and/or there is evidence of an alternative cause such as concurrent medication or illness.

7.4 Recording and Reporting AEs and SAEs

All AEs and SAEs must be recorded in the appropriate CRF, whether or not they are associated to be causally related to study treatment. Each SAE must be reported promptly on the Serious Adverse Event Report Form, and submitted to the Independent Ethics Committee within 24 hours by the investigator. The information recorded on the Serious Adverse Event Report Form will include at least the following: subject number, identity of the event, study drug name and dose, investigator's assessment of the event's severity and relationship to study treatment, and investigator's name and signature. Clinical monitors must collect and verify detailed information of AEs and SAEs when examining original medical records. All AEs and SAEs should be followed up until resolved.

8. Rules of Withdrawal

8.1 Subjects withdraw from the Stud

Subjects can withdraw from the study at any time for any reason without impact on the investigator’s right to treat disease for subjects. Based upon the interest of subjects, the investigator has the right to request subjects to withdraw from the study for any reason including concomitant disease, AEs or treatment failure. The core group of clinical study reserves the right to request subjects to withdraw from the

study for deviation(s) from the protocol, administrative reasons, or other effective or ethical reasons.

The last assessment for subjects must be performed and documented in the CRF regardless of the time and reason for withdrawal. The reason for withdrawal from study participation must be documented in the CRF. All documents related to subjects should be completed. Despite withdrawal from the study, those subjects should be followed up and documented about their diseases until withdrawal of informed consents.

For subjects who withdraw from the study due to concomitant diseases or AEs, the details must be documented in the CRF with other appropriate and valuable data attached.

8.2 Premature Termination of the Study

Reasons for premature termination of the study include external events, repetition of SAEs, growing incidence of treatment-related death and slow enrolment in the study. All subjects will be informed of premature termination of the study by written consents. Any subjects who decide to discontinue participating in the study must report to the principal investigator.

9. Roles of Follow-Up

9.1 Follow-up period

Starting from enrollment.

9.2 Visit Scheduling

Every week for the first three months after enrollment, and then every month until the study is completed.

9.3 Contents

The contents of every follow-up visit include complaints of subjects, vital signs, physical examination, clinical symptoms and clinical laboratory evaluations (hematology, serum chemistry, urinalysis, electrocardiogram, chest imaging examination, and BM assessment). All of the results must be documented in the original medical record.

10. Data Analysis and Statistical Considerations

10.1 Sample Size Assumptions

The sample size was calculated according to the primary endpoint (ORR) of the study. Our previous study reported that the ORR of patients with R/R AML treated with the CDCAG regimen was 46.2% [22]. Additionally, several clinical trials revealed that the ORRs for adult patients with R/R AML receiving venetoclax along with intensive chemotherapy (FLAG-IDA) were 72–75% [23]. Therefore, an expected ORR of 75% was established for patients treated with the CACAG+VEN regimen. A sample size of 27 achieves 90% power to detect differences using a two-sided exact test with a significance level (alpha) of 0.05 (PASS software, NCSS LCC, USA). Allowing a drop-out rate of 20%, 34 patients were required.

10.2 Primary Efficacy Endpoint

The primary endpoint was the treatment-related adverse events and ORR after one cycle of CACAG+VEN treatment.

10.3 Secondary Efficacy Endpoints

The secondary endpoints are CRc rate, PR rate, NR rate, MRD negative rate, OS, PFS, DOR and CIR.

10.4 Data Analysis Considerations

10.4.1 Analysis Population

The primary population will be the ITT population, which is defined as all subjects enrolled to the CACAG+VEN treatment. This ITT population will be the basis for the analysis of efficacy and safety endpoints in this study.

10.4.2 Analysis Plan

10.4.2.1 Baseline Data

Baseline characteristics will be summarized and described in a frequency list.

10.4.2.2 Analysis of Efficacy

The definition of efficacy endpoints has been detailed in previous section. The patient status was determined through telephone follow-ups and the review of outpatient or inpatient records. Continuous data are described as the median with range or mean and standard deviation (SD) according to the normality of the distribution. Categorical data are described as n (%). The ORR, CR, CRi, CRc, and MRD-negative rates were calculated using 95% confidence interval (CI). The Kaplan-Meier method was used to estimate the DOR, PFS, and OS. The cumulative incidence of relapse was estimated using a competing risk model. Death without relapse was defined as a competing event for relapse. Safety analysis was used to calculate the frequency of various events and the ratio of the severity of each event by detailing the cases of blood toxic or nonblood toxic reactions. A two-sided P value < 0.05 was considered to indicate statistical significance. All statistical analyses were performed using Easy R (version 1.61) and R (version 4.3.3).

10.4.2.3 Analysis of Safet

AEs are recorded up to 4 weeks after the discontinuation of the trial therapy, and were graded according to the National Cancer Institute CTCAE version 4.0. The study adjudication committee (consisting of experts in hematology, infection, pathology, pharmacy, and statistics) judge whether adverse events are treatment-related or non-treatment-related. SAEs are those that resulted in death, disability, or incapacity, are life-threatening or judged an important medical event, or required hospitalization or prolongation of existing hospitalization.

11. Materials for the Study

All materials will be provided to study sites and investigators are as follows:
(1) The study protocol;
(2) Informed consent;
(3) CRF.

12. Ethical Considerations

12.1 Responsibilities of Investigators

The investigators have the responsibility for guarantee of the clinical study's compliance with the protocol, Chinese good clinical practice (GCP) guidelines and applicable laws and regulations.

12.2 Informed Consent Process

Prior to participation in the study, subjects must be informed about objectives, methods, possible benefits, potential risks and possible discomforts of the study by investigators. They also should be informed that participation in the study would be voluntary, they can withdraw from the study at any time, there is no impact on the

treatment of the disease whether they take part in the study and their privacy will be protected.
Subjects or their legally acceptable representative should have enough time to read the inform consent and raise queries. Written informed consent must be obtained from each subject, or their legally acceptable representative.

12.3 Good Clinical Practice

This study will be conducted in accordance with the Declaration of Helsinki and Chinese GCP. The study will be conducted only if it is approved by the ethical review committee of the principal study site. The investigators will guarantee that the study will be conducted in accordance with applicable laws and regulations, scientific and ethical principles of the People's Republic of China. If the protocol needs revision during the study, the revised version must be reapproved by the ethical review committee of the principal study site. If new data related to study treatment are discovered, the informed consent must be revised and the revision must be reapproved by the ethical review committee of the principal study site and subjects.

12.4 Protection of Subjects' Personal Data

Data collected in the study are limited to the efficacy and safety related to study treatment. Data will be collected and used in accordance with applicable laws and regulations.

13. Administrative Requirements

Neither the investigator nor the applicant can revise the protocol without agreement of the opposite side. All revisions of the protocol must be released by the applicant institution. To insure the integrity, accuracy and reliability of the data, relevant results of examination and treatment must be documented in original medical record and CRF. Independent clinical monitoring is performed regularly by a panel of

qualified and experienced study investigators composed of hematologists who are blinded as to the treatment assignments.

14. References

[1]. N Bejanyan, DJ Weisdorf, BR Logan, HL Wang, SM Devine, M de Lima, et al.Survival of patients with acute myeloid leukemia relapsing after allogeneic hematopoietic cell transplantation: a center for international blood and marrow transplant research study.Biol Blood Marrow Transplant.2015;3:454-9.

[2]. JM Brandwein, L Saini, MN Geddes, D Yusuf, F Liu, K Schwann, et al.Outcomes of patients with relapsed or refractory acute myeloid leukemia: a population-based real-world study.Am J Blood Res.2020;4:124-133.

[3]. H Dohner, AH Wei, FR Appelbaum, C Craddock, CD DiNardo, H Dombret, et al.Diagnosis and management of AML in adults: 2022 recommendations from an international expert panel on behalf of the ELN.Blood.2022;12:1345-1377.

[4]. RB Walter, M Othus, AK Burnett, B Lowenberg, HM Kantarjian, GJ Ossenkoppele, et al.Resistance prediction in AML: analysis of 4601 patients from MRC/NCRI, HOVON/SAKK, SWOG and MD Anderson Cancer Center.Leukemia.2015;2:312-20.

[5]. F Thol, RF Schlenk, M Heuser and A GanserHow I treat refractory and early relapsed acute myeloid leukemia.Blood.2015;3:319-27.

[6]. S Caruso, B De Angelis, F Del Bufalo, R Ciccone, S Donsante, G Volpe, et al.Safe and effective off-the-shelf immunotherapy based on CAR.CD123-NK cells for the treatment of acute myeloid leukaemia.J Hematol Oncol.2022;1:163.

[7]. X Jin, M Zhang, R Sun, H Lyu, X Xiao, X Zhang, et al.First-in-human phase I study of CLL-1 CAR-T cells in adults with relapsed/refractory acute myeloid leukemia.J Hematol Oncol.2022;1:88.

[8]. X Yang and J WangPrecision therapy for acute myeloid leukemia.J Hematol Oncol.2018;1:3.

[9]. JM Adams and S CoryThe Bcl-2 apoptotic switch in cancer development and therapy. Oncogene.2007;9:1324-37.
[10]. M Konopleva, S Zhao, W Hu, S Jiang, V Snell, D Weidner, et al.The anti-apoptotic genes Bcl-X(L) and Bcl-2 are over-expressed and contribute to chemoresistance of non-proliferating leukaemic CD34+ cells.Br J Haematol.2002;2:521-34.
[11]. R Buettner, LXT Nguyen, C Morales, MH Chen, X Wu, LS Chen, et al.Targeting the metabolic vulnerability of acute myeloid leukemia blasts with a combination of venetoclax and 8-chloro-adenosine.J Hematol Oncol.2021;1:70.
[12]. JM Bogenberger, D Delman, N Hansen, R Valdez, V Fauble, RA Mesa and R TibesEx vivo activity of BCL-2 family inhibitors ABT-199 and ABT-737 combined with 5-azacytidine in myeloid malignancies.Leuk Lymphoma.2015;1:226-9.
[13]. CD DiNardo, A Maiti, CR Rausch, N Pemmaraju, K Naqvi, NG Daver, et al.10-day decitabine with venetoclax for newly diagnosed intensive chemotherapy ineligible, and relapsed or refractory acute myeloid leukaemia: a single-centre, phase 2 trial.Lancet Haematol.2020;10:e724-e736.
[14]. CD DiNardo, K Pratz, V Pullarkat, BA Jonas, M Arellano, PS Becker, et al.Venetoclax combined with decitabine or azacitidine in treatment-naive, elderly patients with acute myeloid leukemia.Blood.2019;1:7-17.
[15]. CD DiNardo, KW Pratz, A Letai, BA Jonas, AH Wei, M Thirman, et al.Safety and preliminary efficacy of venetoclax with decitabine or azacitidine in elderly patients with previously untreated acute myeloid leukaemia: a non-randomised, open-label, phase 1b study.Lancet Oncol.2018;2:216-228.
[16]. I Aldoss, D Yang, A Aribi, H Ali, K Sandhu, MM Al Malki, et al.Efficacy of the combination of venetoclax and hypomethylating agents in relapsed/refractory acute myeloid leukemia.Haematologica.2018;9:e404-e407.
[17]. I Aldoss, D Yang, R Pillai, JF Sanchez, M Mei, A Aribi, et al.Association of leukemia genetics with response to venetoclax and hypomethylating agents in relapsed/refractory acute myeloid leukemia.Am J Hematol.2019;10:E253-e255.

[18]. AH Wei, P Montesinos, V Ivanov, CD DiNardo, J Novak, K Laribi, et al.Venetoclax plus LDAC for newly diagnosed AML ineligible for intensive chemotherapy: a phase 3 randomized placebo-controlled trial.Blood.2020;24:2137-2145.

[19]. M Hong, H Zhu, Q Sun, Y Zhu, Y Miao, H Yang, et al.Decitabine in combination with low-dose cytarabine, aclarubicin and G-CSF tends to improve prognosis in elderly patients with high-risk AML.Aging (Albany NY).2020;7:5792-5811.

[20]. L Dou, Q Xu, M Wang, Y Xiao, L Cheng, H Li, et al.Clinical efficacy of decitabine in combination with standard-dose cytarabine, aclarubicin hydrochloride, and granulocyte colony-stimulating factor in the treatment of young patients with newly diagnosed acute myeloid leukemia.Onco Targets Ther.2019;5013-5023.

[21]. H Wang, YC Liu, CY Zhu, F Yan, MZ Wang, XS Chen, et al.Chidamide increases the sensitivity of refractory or relapsed acute myeloid leukemia cells to anthracyclines via regulation of the HDAC3 -AKT-P21-CDK2 signaling pathway.J Exp Clin Cancer Res.2020;1:278.

[22]. L Wang, J Luo, G Chen, M Fang, X Wei, Y Li, et al.Chidamide, decitabine, cytarabine, aclarubicin, and granulocyte colony-stimulating factor (CDCAG) in patients with relapsed/refractory acute myeloid leukemia: a single-arm, phase 1/2 study.Clin Epigenetics.2020;1:132.

[23]. CD DiNardo, CA Lachowiez, K Takahashi, S Loghavi, L Xiao, T Kadia, et al.Venetoclax Combined With FLAG-IDA Induction and Consolidation in Newly Diagnosed and Relapsed or Refractory Acute Myeloid Leukemia.J Clin Oncol.2021;25:2768-2778.

[24]. BD Cheson, JM Bennett, KJ Kopecky, T Büchner, CL Willman, EH Estey, et al.Revised recommendations of the International Working Group for Diagnosis, Standardization of Response Criteria, Treatment Outcomes, and Reporting Standards for Therapeutic Trials in Acute Myeloid Leukemia.J Clin Oncol.2003;24:4642-9.
