# Supplementary material for: Phase 1 study of chidamide in combination with venetoclax, azacitidine, aclarubicin, cytarabine and G-CSF for refractory/relapsed acute myeloid leukemia: clinical safety, efficacy, and correlative analysis
Source: Front Immunol. 2025 Dec 11;16:1698710. doi: 10.3389/fimmu.2025.1698710 (PMC12738368; doi:10.3389/fimmu.2025.1698710)
Supplement: Supplementary file 2 [file Table1.docx]

**Supplementary Table 1**

| Inclusion Criteria:   1. Voluntary participation in the clinical study; the patient or their legal   guardian fully understands and is informed of the study and signs the informed consent form (ICF); is willing to follow and complete all of the trial procedures.   1. Patients aged 14–75 years with refractory or relapsed (R/R) AML (not   acute promyelocytic leukemia), diagnosed in accordance with the 2021 edition of the CMA criteria.   1. Liver function: serum aspartate aminotransferase or alanine   aminotransferase ≤ 2.5 × upper limit of normal range (ULN), serum bilirubin ≤ 2 × ULN.   1. Kidney function: serum creatinine ≤ ULN. 2. No uncontrollable infections or serious psychiatric disorders. 3. Eligible patients were required to have an ECOG performance status ≤ 2. |
| --- |
| Exclusion Criteria   1. Pregnant or lactating. 2. Patients with chronic alcohol abuse that affects evaluation of the test results. 3. Patients with mental illness or other conditions that prevent informed consent to complete the treatment and examination steps. 4. Patients less than 6 weeks after surgery on a vital organs of the body. |

**Supplementary Table 2 Patient demographics**

| Pt# | Age (y) | Sex | Disease status | ECOG | FAB | 2022 ELN risk status | Molecular | Baseline parameters | | | | Prior therapy  lines | Prior therapy regimen |
| --- | --- | --- | --- | --- | --- | --- | --- | --- | --- | --- | --- | --- | --- |
|  |  |  |  |  |  |  |  | WBC | Hb | Plt | BM  Blasts (%) |  |  |
| 1 | 74 | M | Refractory | 2 | M2 | Adverse | SRSF2/ASXL1/RUNX1/STAG2/CREBBP | 2.76 | 67 | 10 | 59.0 | 1 | AZA+CAG(1) |
| 2 | 38 | M | Refractory | 2 | M0 | Adverse | FLT3-ITD/SETD2/ZBTB7A | 0.83 | 74 | 197 | 77.0 | 3 | IDA/CAG/SKL1028 |
| 3 | 61 | M | Refractory | 2 | M2 | Favorable | DNMT3A/KIT | 1.3 | 71 | 48 | 47.5 | 1 | VEN+AZA |
| 4 | 70 | M | Refractory | 2 | M4 | Adverse | BCOR/DNMT31/GATA2/NRAS/  ASXL1/NF1 | 3.28 | 68 | 16 | 14.8 | 1 | VEN+AZA+Ara-c |
| 5 | 43 | M | Refractory | 2 | M5b | Adverse | / | 15.26 | 72 | 23 | 78.0 | 3 | AZA+HA/AZA+Aclarubicin+HAG/VDAP+Cladribine |
| 6 | 33 | M | Refractory | 1 | M2 | Adverse | TP53/WT1 | 3.54 | 80 | 16 | 13.0 | 3 | IDA/AZA+HDAC/VEN+AZA |
| 7 | 41 | F | Early relapse | 2 | M0 | Adverse | KIT/IDH2/ASXL1 | 17.2 | 57 | 21 | 91.6 | 15 | IDA/IDA+Decitabine/HDAC(2)/AZA+HAG/AZA+CAG+PD1/VEN+PD1/VEN+AZA+Selinexor/FLAG(3)/AZA+CAG+PD1/AZA+VEN+Ara-c/AZA+VEN+Selinexor |
| 8 | 15 | M | Early relapse | 2 | M5b | Adverse | KMT2A/NRAS | 18.41 | 124 | 34 | 70.0 | 3 | IDA+VP16+Luxolitinib/HDAC+Chidamide/HDAC |
| 9 | 52 | F | Early relapse | 1 | M4 | Favorable | Biallelic CEBPA/TET2 | 2.58 | 94 | 132 | 34.8 | 4 | Decitabine+IDA/HDAC(3) |
| 10 | 42 | F | Late relapse | 1 | M5b | Favorable | / | 2.58 | 94 | 132 | 34.8 | 7 | DA/HDAC(5)/MA |
| 11 | 32 | F | Early relapse | 2 | M2 | Intermediate | / | 2.16 | 90 | 65 | 77.2 | 6 | IDA/VEN+AZA+CAG(5) |
| 12 | 59 | F | Early relapse | 1 | M4 | Intermediate | FLT3-TKD/STAG2/SRSF2/NRAS/KRAS | 6.28 | 95 | 62 | 6.8 | 3 | VEN+AZA/HVA/HDAC |
| 13 | 58 | M | Early relapse | 1 | M2 | Intermediate | DNMT3A/PHF6 | 2.51 | 81 | 39 | 26.0 | 7 | IDA/AZA(3)/HDAC(2)/TA |
| 14 | 35 | F | Early relapse | 1 | M5 | Adverse | FLT3-ITD/KIT | 15.18 | 78 | 6 | 84.4 | 2 | IDA(2) |
| 15 | 27 | F | Late relapse | 1 | M5 | Intermediate | IDH1/NRAS | 2.5 | 110 | 154 | 17.2 | 6 | DA(2)/HDAC(3)/AA |
| 16 | 19 | M | Early relapse | 1 | M5 | Adverse | FLT3-ITD/TET2/TP53 | 5.68 | 69 | 30 | 44.8 | 2 | IDA/HDAC |
| 17 | 31 | F | Late relapse | 2 | M4 | Intermediate | DNMT3A/FLT3-TKD/GATA2/IDH1/MYC | 2.36 | 96 | 77 | 30.0 | 2 | IDA/DCIG |
| 18 | 46 | M | Refractory | 2 | M4 | Adverse | / | 0.63 | 74 | 114 | 89.0 | 1 | IDA |
| 19 | 33 | F | Refractory | 2 | M4 | Favorable | NRAS/DNMT3/IDH1/NPM1/KRAS | 4.02 | 94 | 316 | / | 2 | IDA/AZA+VEN+Chidamide |
| 20 | 39 | F | Late relapse | 1 | M2 | Favorable | Biallelic-CEBPA/ASXL1 | 4.38 | 115 | 64 | 44.0 | 7 | IDA(2)/IAE/HDAC(4) |
| 21 | 45 | F | Refractory | 1 | M5 | Adverse | FLT3-ITD/RUNX1/KMT2A/DDX41 | 1.63 | 73 | 42 | 43.0 | 1 | DDAG |
| 22 | 30 | M | Late  relapse | 1 | M2 | Intermediate | / | 1.28 | 52 | 9 | 10.0 | 17 | CAG(2)/TA(6)/IDA(2)/Decitabine+HAA(3)/Decitabine+daunorubicin+HA(2)/AZA+VP-16+HA/IDA+HDAC |
| 23 | 65 | M | Refractory | 2 |  | Intermediate | / | 2.49 | 56 | 143 | 12.0 | 1 | VEN+Alarubicin+Ara-c |
| 24 | 16 | M | Refractory | 1 | M4 | Adverse | KRAS/BCOR | 1.54 | 71 | 16 | 6.4 | 1 | DA |
| 25 | 57 | M | Refractory | 2 | M2/M5 | Intermediate | / | 2.81 | 45 | 92 | 60.4 | 1 | HAA+Ara-c |
| 26 | 59 | M | Refractory | 2 |  | Adverse | TP53 | 2.91 | 62 | 36 | 11.6 | 1 | AZA+VEN+Decitabine |
| 27 | 54 | F | Refractory | 1 | M5b | Adverse | TP53 | 30.17 | 71 | 21 | 93.2 | 2 | DA(1)/Lenalidomide+VEN |
| 28 | 46 | F | Early relapse | 1 |  | Adverse | IDH1/IDH2/WT1 | 0.97 | 72 | 12 | 4.4 | 4 | IDA(2)/AZA+VEN/DA |
| 29 | 67 | M | Early relapse | 1 |  | Intermediate | / | 142.82 | 56 | 41 | 88.0 | 4 | AZA+VEN(2)/AZA/AZA+Ara-c |
| 30 | 58 | F | Refractory | 1 | M2a | Adverse | IDH2/TET2/BCOR/CREBBP/DNMT3A/IGLL5 | 1.3 | 81 | 90 | 38.0 | 1 | DA |
| 31 | 52 | M | Late  relapse | 1 |  | Adverse | KIT/PTPN11 | 9.07 | 150 | 47 | 63.3 | 11 | MA(2)/HDAC(2)/DCAG/HDAC(2)/Decitabine+G-CSF+Ara-C(2)/AZA/AZA+VEN |
| 32 | 62 | M | Refractory | 0 |  | Adverse | ASXL1 | 3.61 | 114 | 119 | 11.2 | 11 | AZA+HA(5)/AZA+CAG(2)/AZA+DA(3)/AZA+AA |
| 33 | 42 | M | Late  relapse | 2 |  | Intermediate | / | 2.22 | 95 | 10 | 26.4 | 1 | Allo-HSCT |
| 34 | 49 | F | Refractory | 2 | M5 | Favorable | DNMT3A/NPM1/IDH2/ZFHX4/TYK2/ARIDIB | 0.88 | 90 | 119 | 30.4 | 1 | DA |

Abbreviations:AZA: Azacitidine, VEN: Venetoclax, VP16: Etoposide, CAG: Cytarabine, aclarubicin and G-CSF, IDA: Idarubicin and cytarabine,HA: [Homoharringtonine](https://www.medchemexpress.cn/Homoharringtonine.html) and cytarabine, HAG: [Homoharringtonine](https://www.medchemexpress.cn/Homoharringtonine.html), cytarabine and G-CSF, VDAP: Vincristine, daunorubicin, cytarabine and prednisone,HDAC: High dose Ara-C, LDAC: Low dose Ara-C, DA: Daunorubicin and cytarabine, MA: Mitoxantrone liposomal and cytarabine, HVA: [Homoharringtonine](https://www.medchemexpress.cn/Homoharringtonine.html),venetoclax and azacitidine, TA: Pirarubicin and cytarabine, AA: Aclarubicin and cytarabine, DCIG: Decitabine, idarubicin, cytarabine and G-CSF,IAE: Idarubicin, cytarabine and etoposide, DDAG:Decitabine, cytarabine, daunorubicin and G-CSF, HAA; [Homoharringtonine](https://www.medchemexpress.cn/Homoharringtonine.html), aclarubicin and cytarabine, DCAG: Decitabine, aclarubicin, cytarabine and G-CSF

**Supplementary Table 3 Responses after one cycle of CACAG-VEN therapy in patients who received different chemotherapy cycles (n = 34)**

|  | Chemotherapy cycles before CACAG-VEN | | | |
| --- | --- | --- | --- | --- |
|  | 0 | 1 cycle | 2 cycles | ≥ 3 cycles |
| Clinical response, n (%, 95% CI) | 14 | 13 | 3 | 4 |
| ORR | 12 (85.7, 57.2–98.2) | 9 (69.2, 38.6–90.9) | 3 (100, 29.2–100) | 2 (50, 6.8–93.2) |
| CRc | 12 (85.7, 57.2–98.2) | 9 (69.2, 38.6–90.9) | 2 (66.7, 9.4–99.2) | 2 (50, 6.8–93.2) |
| CRc _MRD+_ | 5 (41.7, 15.2–72.3) | 7 (77.8, 40.0–97.2) | 1 (50, 1.3–98.7) | 1 (50, 1.3–98.7) |
| CRc _MRD-_ | 7 (58.3, 27.7–84.8) | 2 (22.2, 2.8–60.0) | 1 (50, 1.3–98.7) | 1 (50, 1.3–98.7) |
| PR | 0 (0, 0.0–23.2) | 0 (0, 0.0–24.7) | 1 (33.3, 0.8–90.6) | 0 (0, 0.0–60.2) |
| NR | 2 (14.3, 1.8–42.8) | 4 (30.8, 9.1–61.4) | 0 (0, 0.0–70.8) | 2 (50, 6.8–93.2) |

**Supplementary Table 4 Responses after one cycle of CACAG-VEN therapy in patients who received different chemotherapy regimens (n = 34)**

|  | Regimens before CACAG-VEN | | | | | | |
| --- | --- | --- | --- | --- | --- | --- | --- |
|  | 3+7 | 3+7 only* | Azaciitidine | Decitabine | VEN | VEN+HMAs | Chidamide |
| Clinical response, n (%, 95% CI) | 26 | 11 | 17 | 7 | 12 | 12 | 4 |
| ORR | 22 (84.6, 65.1–95.6) | 11 (100, 71.5–100) | 10 (58.8, 32.9–81.6) | 5 (71.4, 29.0–96.3) | 7 (58.3, 27.7–84.8) | 6 (50, 21.1–78.9) | 4 (100, 39.8–100) |
| CRc | 19 (73.1, 52.2–88.4) | 11 (100, 71.5–100) | 10 (58.8, 32.9–81.6) | 5 (71.4, 29.0–96.3) | 6 (50, 21.1–78.9) | 6 (50, 21.1–78.9) | 4 (100, 39.8–100) |
| CR _MRD+_ | 8 (42.1, 20.3–66.5) | 5 (45.5, 16.7–76.6) | 6 (60, 26.2–87.8) | 3 (60, 14.7–94.7) | 5 (83.3, 35.9–99.6) | 5 (83.3, 35.9–99.6) | 2 (50, 6.8–93.2) |
| CR _MRD-_ | 11 (57.9, 33.5–79.7) | 6 (54.5, 23.4–83.3) | 4 (40, 12.2–73.8) | 2 (40, 5.3–85.3) | 1 (16.7, 0.5–64.1) | 1 (16.7, 0.5–64.1) | 2 (50, 6.8–93.2) |
| PR | 3 (11.5, 2.4–30.2) | 0 (0, 0.0–28.5) | 0 (0, 0.0–19.5) | 0 (0, 0.0–41.0) | 1 (8.3, 0.2–38.5) | 0 (0, 0.0–28.5) | 0 (0, 0.0–60.2) |
| NR | 4 (15.4, 4.4–34.9) | 0 (0, 0.0–28.5) | 7 (41.2, 18.4–67.1) | 2 (28.6, 3.7–71.0) | 5 (41.7, 15.2–72.3) | 6 (50, 21.1–78.9) | 0 (0, 0.0–60.2) |

*Prior “7+3” only: prior used "7+3" regimen without venetoclax/azacitidine/decitabine/chidamide

**Supplementary Table 5 Responses after one cycle of CACAG-Venetoclax therapy by 2022 ELN risk status (n = 34)**

|  | 2022 ELN risk status | | |
| --- | --- | --- | --- |
|  | Favorable | Intermediate | Adverse |
| Clinical response, n (%, 95% CI) | 6 | 10 | 18 |
| ORR | 6 (100, 54.1–100) | 7 (70, 34.8–93.3) | 13 (72.2, 46.5–90.3) |
| CRc | 6 (100, 54.1–100) | 7 (70, 34.8–93.3) | 12 (66.7, 41.0–86.7) |
| CRc _MRD+_ | 2 (33.3, 4.3–77.7) | 4 (57.1, 18.4–90.1) | 8 (66.7, 34.9–90.1) |
| CRc _MRD-_ | 4 (66.7, 22.3–95.7) | 3 (42.9, 9.9–81.6) | 4 (33.3, 9.9–65.1) |
| PR | 0 (0, 0.0–45.9) | 0 (0, 0.0–30.8) | 1 (5.6, 0.1–27.3) |
| NR | 0 (0, 0.0–45.9) | 3 (30, 6.7–65.2) | 5 (27.8, 9.7–53.5) |

**Supplementary Table 6 Summary of sequenced cells per sample**

| Sample | Sequenced | PassedQC | Tumor/  HSC | GMP/  proMono | Monocyte/  macrophage | Neutrophil | Lymphocyte | Megakaryocyte |
| --- | --- | --- | --- | --- | --- | --- | --- | --- |
| PR#post | 4769 | 4769 | 271 | 510 | 748 | 1615 | 1584 | 41 |
| PR#pre | 3362 | 3362 | 830 | 216 | 50 | 481 | 1776 | 9 |
| NR#post | 8916 | 8000 | 3762 | 1058 | 251 | 1012 | 1875 | 42 |
| NR#pre | 11531 | 8000 | 6095 | 762 | 172 | 384 | 573 | 14 |
| CR.1#post | 11678 | 8000 | 1155 | 919 | 957 | 3505 | 1336 | 128 |
| CR.1#pre | 6803 | 6803 | 4052 | 1611 | 29 | 350 | 759 | 2 |
| CR.2#post | 5375 | 5375 | 447 | 234 | 2072 | 1481 | 1009 | 132 |
| CR.2#pre | 10097 | 8000 | 90 | 301 | 1025 | 5339 | 1221 | 24 |

**Supplementary Table 7 Causality Assessment of Selected Serious Adverse Events (SAEs) Using the Naranjo Algorithm**

| Patient Identifier | Adverse Event (Grade) | Outcome | Temporal Relationship to CACAG-VEN | Naranjo Score* | Causality Assessment | Rationale |
| --- | --- | --- | --- | --- | --- | --- |
| Patient 4 | Infectious Shock (Grade 5) | Death (Day 19) | Occurred 4 days after completing a 14-day course of VEN. | 0 | Doubtful | The fatal infection originated from a pre-existing bloodstream infection; it was a consequence of the patient's underlying immunocompromised state due to relapsed/refractory AML—a population at a baseline high risk for life-threatening infections. The event timeline, while coinciding with the treatment course, is consistent with the natural history of the disease. |
| Cohort Summary | Neutropenia (Grade 3/4) | Median duration: 17 days | Throughout and following each treatment cycle | 7 | Probable | This is a known, dose-limiting, and predictable class effect of all components of the regimen (VEN and chemotherapy). The high incidence and prolonged duration are consistent with the regimen's intended intensive myelosuppressive mechanism of action. |
| Cohort Summary | Thrombocytopenia | Median duration: 24 days | Throughout and following each treatment cycle | 7 | Probable | As with neutropenia, this is a direct and expected consequence of the regimen's high-intensity myelosuppression, supported by the high transfusion requirements (median 6.5 platelet units). |
| Representative Cases | Pneumonia (Grade 3/4) | Resolved with therapy | Variable | 0 | Doubtful | These infectious originated from a pre-existing infection; it was a consequence of the patient's underlying immunocompromised state due to relapsed/refractory AML—a population at a baseline high risk for life-threatening infections. The event timeline, while coinciding with the treatment course, is consistent with the natural history of the disease. |
| Representative Cases | Electrolyte Abnormalities (e.g., Hyponatremia, Hypokalemia) (Grade 3/4) | Resolved with supplementation | Variable | 3 | Possible | These can be sequelae of infection, sepsis, poor oral intake, or tumor-related effects. While certain chemotherapeutic agents can cause electrolyte wasting, the direct attribution to the study regimen in this complex patient population is less certain. |

*Naranjo Algorithm Score Interpretation: ≥9 = Definite; 5-8 = Probable; 1-4 = Possible; 0 = Doubtful.

**Supplementary Table 8 The dose-escalation information**

| **Patient Identifier** | **Type of adverse event reported** | **Grade*** | **Treatment regimen** | **Event that led to**  **discontinuation** |
| --- | --- | --- | --- | --- |
| **Patient 1** | Neutropenia/thrombocytopenia/hypokalemia/  vomiting | **1/1/1/1** | CACAG-7 days VEN | **no** |
| **Patient 2** | Neutropenia/thrombocytopenia/hypokalemia | **1/1/2** | CACAG-7 days VEN | **no** |
| **Patient 3** | Neutropenia/thrombocytopenia | **1/1** | CACAG-7 days VEN | **no** |
| **Patient 4** | Neutropenia/thrombocytopenia /Infectious shock | **1/1/5** | CACAG-14 days VEN | **no** |
| **Patient 5** | Neutropenia/thrombocytopenia/liver enzymes/hypokalemia | **1/1/2/1** | CACAG-14 days VEN | **no** |
| **Patient 6** | Neutropenia/thrombocytopenia /pneumonia/liver enzymes/hypokalemia/hypocalcemia | **1/1/2/2/1/1** | CACAG-14 days VEN | **no** |
| **Patient 7** | Neutropenia/thrombocytopenia/liver enzymes/hypokalemia | **1/1/1/2** | CACAG-14 days VEN | **no** |
| **Patient 8** | Neutropenia/thrombocytopenia/hypoalbuminemia/hyponatremia/hypokalemia/hypocalcemia | **1/1/1/1/2/1** | CACAG-14 days VEN | **no** |
| **Patient 9** | Neutropenia/thrombocytopenia /pneumonia/nausea/diarrhea/hypoalbuminemia/hypokalemia | **1/1/2/1/1/1/3** | CACAG-14 days VEN | **no** |
| **Patient 10** | Neutropenia/thrombocytopenia /pneumonia/hypokalemia/hypocalcemia | **1/1/3/2/1** | CACAG-14 days VEN | **no** |
| **Patient 11** | Neutropenia/thrombocytopenia /pneumonia/diarrhea/hypoalbuminemia/hyponatremia/hypocalcemia | **1/1/1/1/1/1/2** | CACAG-14 days VEN | **no** |
| **Patient 12** | Neutropenia/thrombocytopenia /pneumonia/hypokalemia | **1/1/1/2** | CACAG-14 days VEN | **no** |
| **Patient 13** | Neutropenia/thrombocytopenia  /pneumonia/hypoalbuminemia/hypokalemia/hypocalcemia | **1/1/2/1/1/1** | CACAG-14 days VEN | **no** |
| **Patient 14** | Neutropenia/thrombocytopenia/liver enzymes/hypoalbuminemia/hyponatremia | **1/1/1/2/3** | CACAG-14 days VEN | **no** |
| **Patient 15** | Neutropenia/thrombocytopenia/liver enzymes/hypokalemia/hypocalcemia | **1/1/2/3/1** | CACAG-14 days VEN | **no** |
| **Patient 16** | Neutropenia/thrombocytopenia /pneumonia/hypoalbuminemia | **1/1/1/1** | CACAG-14 days VEN | **no** |
| **Patient 17** | Neutropenia/thrombocytopenia/urinary tract hemorrhage/hyponatremia/hypokalemia/hypocalcemia | **1/1/1/3/2/3** | CACAG-14 days VEN | **no** |
| **Patient 18** | Neutropenia/thrombocytopenia /pneumonia/drug-induced kidney injury/hypoalbuminemia/hypokalemia/hypocalcemia | **1/1/3/1/1/2/1** | CACAG-14 days VEN | **no** |
| **Patient 19** | Neutropenia/thrombocytopenia /pneumonia/liver enzymes | **1/1/2/1** | CACAG-14 days VEN | **no** |
| **Patient 20** | Neutropenia/thrombocytopenia /pneumonia/hyponatremia/hypokalemia/hypocalcemia | **1/1/1/3/1/2** | CACAG-14 days VEN | **no** |
| **Patient 21** | Neutropenia/thrombocytopenia /pneumonia/hypokalemia | **1/1/3/3** | CACAG-14 days VEN | **no** |
| **Patient 22** | Neutropenia/thrombocytopenia /pneumonia/hypoalbuminemia/hyponatremia/hypokalemia | **1/1/3/1/1/2** | CACAG-14 days VEN | **no** |
| **Patient 23** | Neutropenia/thrombocytopenia /pneumonia/liver enzymes/hypokalemia | **1/1/1/1/3** | CACAG-14 days VEN | **no** |
| **Patient 24** | Neutropenia/thrombocytopenia/liver enzymes/hypoalbuminemia/hyponatremia/hypokalemia/hypocalcemia | **1/1/1/1/3/2/1** | CACAG-14 days VEN | **no** |
| **Patient 25** | Neutropenia/thrombocytopenia /pneumonia/diarrhea/drug-induced kidney injury/hypoalbuminemia/hypokalemia | **1/1/3/1/2/2/1** | CACAG-14 days VEN | **no** |
| **Patient 26** | Neutropenia/thrombocytopenia/liver enzymes/hypokalemia/hypocalcemia | **1/1/1/2/1** | CACAG-14 days VEN | **no** |
| **Patient 27** | Neutropenia/thrombocytopenia/oral ulcer/hyponatremia/hypokalemia | **1/1/1/2/1** | CACAG-14 days VEN | **no** |
| **Patient 28** | Neutropenia/thrombocytopenia/liver enzymes/hypokalemia/hypocalcemia | **1/1/2/1/1** | CACAG-14 days VEN | **no** |
| **Patient 29** | Neutropenia/thrombocytopenia  /pneumonia/diarrhea/hyponatremia/hypokalemia | **1/1/2/1/1/1** | CACAG-14 days VEN | **no** |
| **Patient 30** | Neutropenia/thrombocytopenia/liver enzymes/urinary tract hemorrhage/hypoalbuminemia/hypokalemia/hypocalcemia | **1/1/1/1/1/1/1** | CACAG-14 days VEN | **no** |
| **Patient 31** | Neutropenia/thrombocytopenia/liver enzymes/hyponatremia/hypocalcemia | **1/1/2/1/1** | CACAG-14 days VEN | **no** |
| **Patient 32** | Neutropenia/thrombocytopenia/liver enzymes/hypokalemia | **1/1/1/1** | CACAG-14 days VEN | **no** |
| **Patient 33** | Neutropenia/thrombocytopenia/urinary tract hemorrhage/hypoalbuminemia/hypokalemia/hypocalcemia | **1/1/1/1/1/2** | CACAG-14 days VEN | **no** |
| **Patient 34** | Neutropenia/thrombocytopenia/liver enzymes/hyponatremia/hypokalemia/hypocalcemia | **1/1/1/1/1/1** | CACAG-14 days VEN | **no** |

*The AE severity grading standard was adopted from the National Cancer Institute (NCI) adverse event grading (CTC-AE) version 4.03.

**Supplementary table 9 Clinical background of patients selected for single-cell sequencing**

| SampleID | Disease status | Time from enrollment | status | Response | Adverse events | Grade* | Outcome | Cause of death |
| --- | --- | --- | --- | --- | --- | --- | --- | --- |
| 1 | Late  relapse | D0 | Pre-treatment | Partial response | Neutropenia/thrombocytopenia /pneumonia/hypoalbuminemia/hyponatremia/hypokalemia | 1/1/3/1/1/2 | Alive | / |
| 2 | Late  relapse | D28 | Post-treatment |  |  |  |  |  |
| 3 | Early relapse | D0 | Pre-treatment | No response | Neutropenia/thrombocytopenia  /pneumonia/diarrhea/hyponatremia/hypokalemia | 1/1/2/1/1/1 | Death | There was no response to treatment in the patient with R/R AML. |
| 4 | Early relapse | D28 | Post-treatment |  |  |  |  |  |
| 5 | Late  relapse | D0 | Pre-treatment | Complete response | Neutropenia/thrombocytopenia/liver enzymes/hyponatremia/hypocalcemia | 1/1/2/1/1 | Alive | / |
| 6 | Late  relapse | D28 | Post-treatment |  |  |  |  |  |
| 7 | Late  relapse | D0 | Pre-treatment | Complete response | Neutropenia/thrombocytopenia/liver enzymes/hyponatremia/hypokalemia/hypocalcemia | 1/1/1/1/1/1 | Alive | / |
| 8 | Late  relapse | D28 | Post-treatment |  |  |  |  |  |

*The AE severity grading standard was adopted from the National Cancer Institute (NCI) adverse event grading (CTC-AE) version 4.03.

**Supplementary Figure 1. Survival analysis**


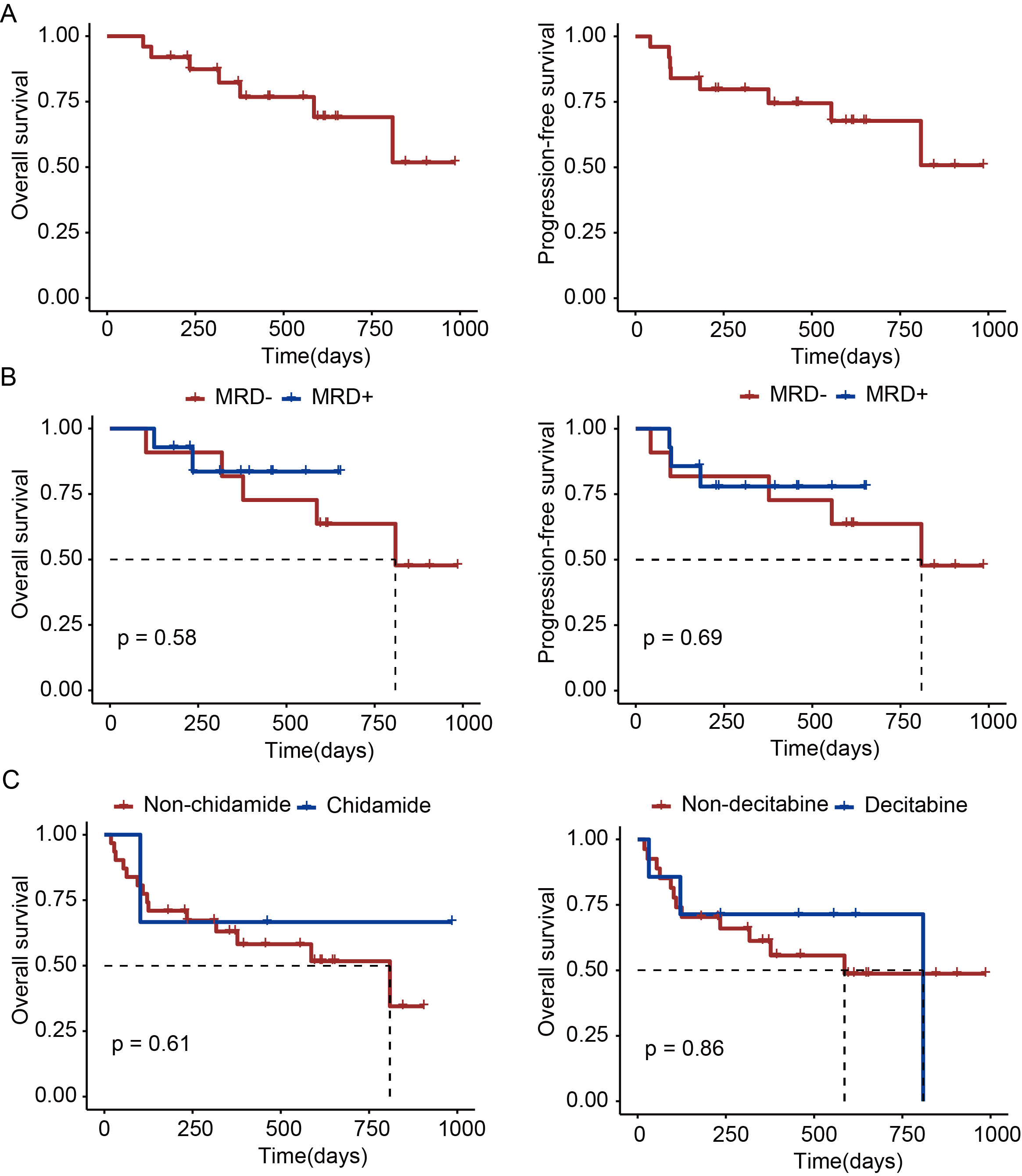


**Supplementary Figure 1. Survival analysis**

(A) Cumulative incidence of overall survival and progression-free survival in patients who achieved CRc. (B) Cumulative incidence of overall and progression-free survival in patients with MRD-negative or not. (C) Cumulative incidence of overall and progression-free survival in patients with prior use of chidamide/decitabine or not.

**Supplementary Figure 2. Single-cell transcriptional landscape of patients pre- and post-CACAG-VEN treatment**

**
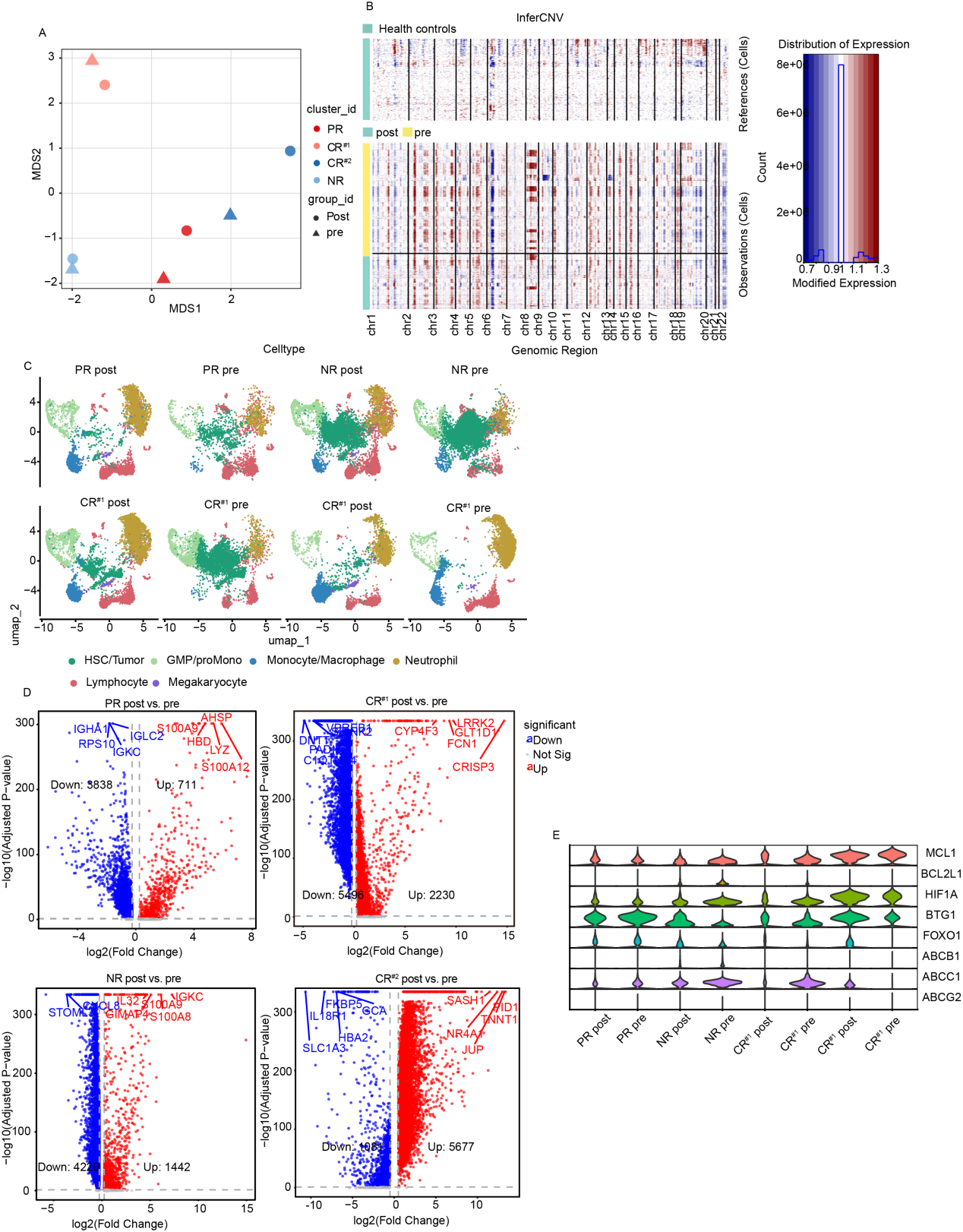
**

**Supplementary Figure 2. Single-cell transcriptional landscape of patients pre- and post-CACAG + VEN treatment**

(A) PCA cluster of eight pre- and post-treatment samples. (B) Heatmap showing the genome-wide inferred CNA profiles for HCs and pre- and post-treatment samples. (C) volcano plot presents the differential expression analysis comparing pre- versus post-treatment genes for each individual patient. (D) Expression of the top 15 shared upregulated differentially expressed genes in NR vs. CR, NR vs. PR. and NR vs. HCs compared to each other. (E) Expression of drug-resistant related genes in each sample.
